# Physiological Age of Potato Seed Tubers of Contrasting Cultivars Hardly Affects Crop Performance in a Temperate Climate

**DOI:** 10.1007/s11540-024-09731-2

**Published:** 2024-05-20

**Authors:** Chunmei Zou, Peter E. L. van der Putten, Marieke Datema, Leon Mossink, Willemien J. M. Lommen, Paul C. Struik, Martin K. van Ittersum

**Affiliations:** 1 Centre for Crop Systems Analysis, Wageningen University & Research, P.O. Box 430, 6700 AK Wageningen, The Netherlands; 2 Plant Production Systems, Wageningen University & Research, P.O. Box 430, 6700 AK Wageningen, The Netherlands; 3 Present Address: Solynta, 6703 HA Wageningen, The Netherlands

**Keywords:** Canopy development, Cultivar, Emergence, Storage temperature, Vigour, Yield analysis

## Abstract

The physiological age of potato seed tubers influences the subsequent field performance of the crop, conditioning its growth and development. This study investigates the effects of the physiological age of seed tubers on the field performance of four contrasting, currently widely grown cultivars (Agria, Festien, Innovator, Lady Claire) across three sites in the Netherlands over 3 years (Cycles 1—3). Seed tubers were produced annually at a single site under uniform conditions, with variations in physiological age induced by applying different storage temperatures (4, 7, 10, and/or 17 °C). Our previous study revealed varying effects of storage temperature on sprouting behaviour among cultivars, suggesting different rates of ageing. After completing the storage season, in the current study these seed tubers were planted in the field and subsequent crop performance was thoroughly phenotyped. Cultivar-specific effects of storage temperature were evident during early growth stages and when seed tubers were stored at 17 °C in Cycle 3. However, within the storage temperature range of 4 to 10 °C, the influence of storage temperatures was small or inconsistent. Overall, effects on canopy cover, stem and tuber number, yield and quality, and tuber yield explanatory variables were limited. The outcomes suggest that while very high storage temperatures should be avoided in regions with long crop cycles, stringent cold storage regimes are unnecessary to achieve optimal growth vigour in potato seed tubers of the studied cultivars in a temperate climate.

**Supplementary Information:**

The online version contains supplementary material available at 10.1007/s11540-024-09731-2.

## Introduction

The physiological age of a potato (*Solanum tuberosum* L.) seed tuber is defined as the physiological status of the tuber that evolves progressively with increasing chronological age, depending on the growth history and storage conditions (Ewing and Struik 1992). The physiological age of a seed tuber plays an important role in determining subsequent crop performance, influencing tuber yield and quality through its effects on emergence, canopy cover development and duration, number of stems, tuber initiation, bulking rate and duration, the number of tubers per stem, and tuber size distribution, as summarised by Struik and Wiersema (1999). Extensive research into the effects of physiological age on crop growth has predominantly compared the planting of physiologically younger and older seed tubers (Kawakami 1963; Iritani 1968; Perennec and Madec 1980; Reust 1982; Moll 1985; Allen and O’Brien 1986; Vakis 1986; Bodlaender and Marinus 1987; Van Loon 1987; Benz and Fahem 1988; Hay and Hampson 1991; Knowles and Botar 1991, 1992; Struik et al. 2006; Oliveira et al. 2017; Mediouni et al. 2020). Commonly observed patterns indicate that plants developing from physiologically older tubers exhibit earlier emergence, faster initial growth rate, earlier tuber initiation, more stems, reduced foliage, and earlier senescence, making physiologically older seed tubers suitable for shorter crop cycles than physiologically younger seed tubers.

Nevertheless, drawing precise and coherent conclusions regarding the impact of physiological age on field performance remains challenging. First, many proposed physiological age indicators, including the popular methods employing accumulated day-degrees (O’Brien et al. 1983) and physiological age index (Caldiz et al. 2001), lack universality across cultivars, growth histories, and storage regimes (Van Ittersum and Scholte 1992; Jenkins et al. 1993; Coleman 2000; Struik 2006; Johansen et al. 2008). The classification of seed tubers as physiologically ‘young’ or ‘old’ remains highly relative, complicating the comparison of studies. Second, the influence of physiological age on field performance is cultivar-specific and varies based on the cultivar’s rate of ageing and maturity type (Moll 1985; Van Loon 1987; Benz and Fahem 1988; Van Ittersum et al. 1990; Hay and Hampson 1991; Van Ittersum 1993; Struik et al. 2006; Blauer et al. 2013). Most existing literature focuses on relatively old and partially outdated cultivars, necessitating an update in our understanding of the behaviour of currently widely grown cultivars. Moreover, the intricate interaction with environmental factors, such as planting date, weather conditions during different crop growth stages, soil type, and agronomic practices, further complicates the deciphering of the effects of physiological age on field performance (Goodwin et al. 1969; Wurr 1978; Van Loon 1987; Hay and Hampson 1991; Struik et al. 2006).

This study seeks to investigate the effects of the physiological age of seed tubers on field performance, addressing the factors above. Seed tubers of four contrasting and currently widely grown cultivars were produced under uniform conditions. Different storage temperatures were applied as the sole treatment to create variations in physiological age. Our previous work has quantified the ageing process of seed tubers during storage through sprouting behaviour analysis, where storage temperatures induced varying sprouting patterns in cultivars characterised by different rates of ageing (Zou et al. 2024). In this study, these seed tubers were planted at three sites and during three cycles, and crop performance was phenotyped in great detail, including emergence, number of stems, canopy development, number of tubers, tuber yield, and quality (including tuber size distribution and tuber dry matter concentration). Key components of yield were examined through yield formation and yield components analyses. Building on insights from the previous sprouting study, we hypothesised that differences in physiological age as indicated by the sprouting behaviour would translate into differences in growth vigour in the field, development of the crop, and yielding capacity. Meanwhile, development later in the growth cycle and yielding capacity would interact with the growing conditions as determined by year and site.

## Materials and Methods

### Experimental Design

The study was conducted over three cycles (2019/2020, 2020/2021, 2021/2022), each consisting of a seed production season, a storage season, and a crop production (field) season. Seed performance during the storage seasons has been analysed and reported by Zou et al. (2024). The present paper focuses specifically on the crop production seasons, during which in total eight field experiments were carried out: two in Cycle 1 and three in Cycles 2 and 3. Each trial field consisted of 36 plots following a randomised complete block design (RCBD) with four cultivars, three storage temperatures as treatments, and three blocks as replicates. Details of cultivars, storage conditions, and field experimental design are explained below.

#### Cultivar Selection

Four cultivars, i.e. Agria, Festien, Innovator, and Lady Claire (in alphabetical order), were selected based on their importance in different market outlets and contrasting phenological and physiological characteristics. Specifically, Agria has late maturity and is slow ageing, Festien has very late maturity and is fast ageing, Innovator has early maturity and is fast ageing, and Lady Claire has early maturity and is slow ageing (Table 1).

**Table 1:** Cultivar information (from Zou et al. 2024)

Cultivar	Year of release	Breeding company	Crossing parents	Market outlet	Maturity^1^	Dormancy^2^	Rate of ageing^3^
Agria	1985	Agrico BV	QUARTA × SEMLO	Table/fries	5.5	7.5	Slow
Festien	2000	Averis Seeds BV	KARTEL × KA80-1920	Starch	3	7.5	Fast
Innovator	1999	HZPC Holland BV	SHEPODY × RZ84-2580	Fries	7	7	Fast
Lady Claire	1996	Meijer Potato BV	AGRIA × KW78-34–470	Crisps	7.5	7	Slow

^1^Low to high from 1 to 10 indicates late to early maturity

^2^Low to high from 1 to 10 indicates short to long dormancy

^3^Based on Zou et al. (2024)

#### Seed Tuber Production

During each seed production season, seed tubers of all four cultivars were produced at a single field site at the experimental farm of Stichting Proefboerderijen Noordelijke Akkerbouw (SPNA) de Kollumerwaard, in Munnekezijl, the Netherlands (approx. 53°20′ N, 6°15′ E). Seed production followed common and strictly controlled agricultural practices for seed tuber production during the growing season, haulm killing, harvesting, wound healing, and local storage, until the seed tubers were certified (free of *Ralstonia solanacearum* and *Clavibacter michiganensis* subsp. *sepedonicus*) and transported to Wageningen for the storage season. Pre-storage temperature-sums (T-sums) during each cycle are listed in Table 2 after “Seed production until harvest”. To minimise initial differences due to size (Struik and Wiersema 1999), only seed tubers measuring 35—45 mm in Cycle 1 (a slightly larger size range of Agria and Innovator due to a limited number of tubers in 35—40 mm) and 35—40 mm in Cycles 2 and 3 were selected.

**Table 2:** T-sums (°Cd) during the different periods preceding planting of the seed tubers at three sites (Est, Nagele, Wanroij) in three cycles. T-sum is the accumulated daily temperature (mean hourly air temperature above a base temperature of 0 °C). Values shown in parentheses were estimated based on the average daily temperature in the corresponding periods in other cycles (for Cycle 1) or at other sites (for Cycle 3)

Period	Cycle 1		Cycle 2			Cycle 3		
	Est	Wanroij	Est	Nagele	Wanroij	Est	Nagele	Wanroij
Seed production until harvest^1^	1900	1900	1898	1898	1898	1641	1641	1641
Harvest until storage	(1100)	(1100)	808	808	808	1042	1042	1042
Storage								
4 °C	616	616	607	607	607	667	667	667
7 °C	1276	1276	1133	1133	1133	-	-	-
10 °C	1602	1602	1581	1581	1581	1706	1706	1706
17 °C	-	-	-	-	-	2834	2834	2834
Post-storage until planting^2^ (mini-chitting)	(77)	(176)	92	73	176	152	172	(219)
Total								
4 °C	(3693)	(3792)	3405	3386	3489	3502	3522	(3569)
7 °C	(4353)	(4452)	3931	3912	4015	-	-	-
10 °C	(4679)	(4778)	4379	4360	4463	4541	4561	(4608)
17 °C	-	-	-	-	-	5669	5689	(5736)

^1^Planting of mother tubers until harvest

^2^Planting of the seed tubers in the crop production season

#### Storage

To create different physiological ages, seed tubers were stored at different and constant temperatures in darkness. In Cycles 1 and 2, the storage temperatures were 4, 7, and 10 °C. In Cycle 3, the storage temperatures were 4, 10, and 17 °C to create a wider range. Storage T-sums of each temperature treatment across cycles are listed in Table 2 after “Storage” (for details, see Zou et al. 2024). During the storage periods of the three cycles, no instances of rotten tubers, moulded tubers, visual symptoms of diseases, or formation of ‘little potatoes’ were observed.

Sprouting behaviours during storage and in sprouting tests were reported by Zou et al. (2024). At the end of each storage season, all seed tubers had broken endo-dormancy. In the sprouting tests, seed tubers under certain treatments passed their maximum sprouting capacity, indicated by reduced total sprout dry weight per tuber compared to the previous sampling. In contrast, seed tubers under other treatments had not yet reached their maximum sprouting capacity. There was a strong cultivar and storage temperature interaction: Festien and Innovator stored at 17 °C, 10 °C, and in some cases 7 °C passed their maximum, while Agria and Lady Claire did so only when stored at 17 °C (Fig. S1, Zou et al. 2024).

At the end of storage, seed tubers with sprouts ≥ 2 mm were de-sprouted, and all seed tubers were subsequently placed under a semi-open barn for mini-chitting for around 2 weeks (1—3 weeks) until being transported to the trial sites for planting. T-sums during this phase are listed in Table 2 after “Post-storage until planting (mini-chitting)”. Seed tubers’ appearance and fresh weight are shown in Figs. S2 and S3, respectively. Seed tubers’ mineral nutrient contents at planting time in Cycle 3 are listed in Table S1, indicating that storage at 17 °C significantly reduced the content (in mg tuber^−1^) of most of the tested nutrients compared to storage at 4 °C across cultivars.

#### Locations and Field Management

Trial sites were located on commercial farms in different regions of the Netherlands and were named after the nearby village (Table 3). In Cycle 1, there were two sites: Est (central NL, heavy river clay soil) and Wanroij (south-eastern NL, sandy soil). In Cycles 2 and 3, there were three sites: Est and Wanroij (as above) and Nagele (northern NL, loamy soil). The fields were conventionally managed by farmers similar to their other potato fields, including fertilisation and irrigation (if applied) (Table S2). Herbicides were applied 1 week after planting (before emergence), fungicides against late blight were applied weekly after emergence, and insecticides and other fungicides were applied when needed.

**Table 3:** Details of trial fields. Estimated values are shown in parentheses

	Cycle 1		Cycle 2			Cycle 3		
	Est	Wanroij	Est	Nagele	Wanroij	Est	Nagele	Wanroij
Approximate location	51°50′46″ N 5°19′24″ E	51°39′17″ N 5°50′31″ E	51°50′51″ N 5°20′32″ E	52°38′58″ N 5°42′26″ E	51°39′23″ N 5°50′30″ E	51°50′58″ N 5°19′53″ E	52°39′23″ N 5°42′04″ E	51°39′39″ N 5°51′00″ E
Planting date	28/04/2020	07/05/2020	01/05/2021	28/04/2021	07/05/2021	05/05/2022	06/05/2022	09/05/2022
Harvest date	23/09/2020	02/10/2020	29/09/2021	24/09/2021	05/10/2021	30/09/2022	22/09/2022	13/10/2022
Field period (d)	148	148	151	149	151	148	139	157
T-sum (°Cd)^1^	2458	2552	2437	2353	2504	2541	2373	2726
Temperature (°C)^2^	16.6	17.2	16.1	15.8	16.6	17.2	17.1	17.4
Planting arrangement (m × m)	0.75 × 0.27	0.75 × (0.275)	0.75 × 0.28	0.75 × 0.28	0.75 × 0.28	0.75 × 0.31	0.75 × 0.28	0.75 × 0.31
Plant density (plants m^−2^)	4.94	(4.85)	4.76	4.76	4.76	4.30	4.76	4.30

^1^Accumulated daily temperature (mean hourly temperatures above a base temperature of 0 °C)

^2^Average daily air temperature, calculated as T-sum divided by the field period

#### Experimental Layout

Each trial was conducted on an area of approx. 0.3 ha and the trial field was bordered by four rows of farmers’ seed tubers. Each trial consisted of 36 plots and each plot consisted of four rows at a row distance of 0.75 m and 42 (Cycle 1) or 45 (Cycles 2 and 3) seed tubers per row planted at within-row planting distances of 0.27—0.31 m. Only plants in the middle two rows of a plot were used for measurements. Tubers were machine-planted. For details, see Table 3.

#### Weather Conditions

Hourly data on air temperatures, global radiation and precipitation (Figs. S4 and S5) were retrieved from online data (KNMI 2023) of the nearest Koninklijk Nederlands Meteorologisch Instituut (KNMI) weather stations to the field sites (Herwijnen station—Est 13 km, Marknesse station—Nagele 14 km, Volkel station—Wanroij 10 km).

### Data Collection and Analyses

#### Emergence

The number of emerged plants was counted 1 to 2 times per week starting around 3 weeks after planting and until a plot reached 100% emergence or the percentage stopped increasing. An additional final check was performed before canopy closure in Cycle 3. In Cycle 1, the number of emerged plants was counted per plot from an area of 32 planted seed tubers, and the emerged plants were visually estimated to be either emerged today, emerged 2 days ago, or emerged 4 days ago. In Cycle 1 at Wanroij, we stopped after two assessments due to uneven planting distances. In Cycles 2 and 3 at all sites, the number of emerged plants was counted in one of the middle rows (45 planted seed tubers per plot), and the date of emergence was visually estimated to be either expected to emerge tomorrow (visible crack in the soil surface), emerged today, emerged yesterday, emerged 2 days ago, emerged 3 days ago, or emerged 4 days ago.

*Final emergence* was calculated as the number of emerged plants during the last observation as a percentage of the total number of planted seed tubers in the measurement area. The *days after planting (DAP) until 75% emergence* was the days after planting until 75% emergence of the planted seed tubers was reached; it was calculated per plot by linear interpolation between the closest data points below and above (or equal to) 75% emergence.

#### Canopy Cover

Within a fixed area of eight planted seed tubers per plot, the percentage of soil cover by the green canopy (canopy cover) was estimated weekly from emergence until haulm killing (if taken place, see Table S3) or final harvest. Two pictures (one per middle row) were taken per plot per measuring date by a camera hanging 0.97 m above the top of the canopy and in the centre of a tailor-made metal grid of 0.99 m × 0.75 m. Each picture covered around 3.2 (planting distance 0.31 m) to 3.7 (planting distance 0.27 m) plants. The percentage of canopy area was analysed using CANOPEO (Patrignani and Ochsner 2015) in MATLAB Version 9.13.0 (R2022b) (The MathWorks Inc. 2022). The percentage of canopy cover of each plot per assessment date was calculated as the average of the two middle rows.

The daily canopy cover was estimated by fitting the canopy cover against days after planting per combination of cycle × site × cultivar × storage temperature with the model of Khan et al. (2019). The model comprises three phases: the canopy build-up phase *P*1 (Eq. 1), the maximum canopy cover phase *P*2 (Eq. 2), and the canopy decline phase *P*3 (Eq. 3), and in total five parameters (*t*_m1_,* t*_1_,* t*_2_, *t*_e_, and *v*_max_) (Fig. S6):

1 $$v= {v}_{\text{max}} \left(1+\frac{{t}_{1}-t}{{t}_{1}-{t}_{\text{m1}}}\right) \left(\frac{t}{{t}_{1}}\right){ }^{\frac{{t}_{1}}{{t}_{1}-{t}_{\text{m1}}}}\text{ with }0 \le t\le {t}_{1}$$

2 $$v= {v}_{\text{max}} {\text{with}}\; t_{1}\;{<}{t}\;{<}{t}_{2}$$

3 $$v= {v}_{\text{max}} \left(\frac{{t}_{e}-t}{{t}_{e}-{t}_{2}}\right) \left(\frac{t+{t}_{1}- {t}_{2}}{{t}_{1}}\right){ }^{\frac{{t}_{1}}{{t}_{e}-{t}_{2}}} \;\mathrm{with }\;{t}_{2} \le t\le {t}_{e}$$

where *v* (canopy cover, in percentage) is the response variable, *t* (time, in days after planting) is the explanatory variable, *v*_max_ (%) is the parameter indicating the maximum value of canopy cover *v*, *t*_m1_ (*d*) is the inflexion point in the build-up phase *P*1, from where the rate of increase begins to decrease, *t*_1_ (*d*) is the end of the build-up phase *P*1, *t*_2_ (*d*) is the end of the maximum canopy cover phase *P*2 and *t*_e_ (*d*) is the end of the canopy decline phase *P*3.

Subsequently, based on the estimated parameter values, the area under the curve (AUC) of *P*1, *P*2, and *P*3 was calculated by integrating Eqs. 1, 2, and 3, as *A*_1_, *A*_2_, and *A*_3_, respectively, according to Khan et al. (2019):

4 $${A}_{1}={v}_{{\text{ma}}{\text{x}}}\left[\frac{2{t}_{1}({t}_{1}-{t}_{\text{m1}})}{{3t}_{1}-2{t}_{\text{m1}}}\right]$$

5 $${A}_{2}={v}_{\text{max}}({t}_{2}-{t}_{1})$$

6 $${A}_{3}=\frac{{v}_{\text{max}}\left({t}_{e}-{t}_{2}\right)}{2{t}_{e}-2{t}_{2}+{t}_{1}} \, \left[\left({t}_{e}-{t}_{2}+{t}_{1}\right) {\left(\frac{{t}_{e}-{t}_{2}+{t}_{1}}{{t}_{1}}\right)}^{\frac{{t}_{1}}{{t}_{e}-{t}_{2}}}-{2}{t}_{1}\right]$$

When the estimated values of temporal parameters, e.g. *t*_e_, surpassed the actual timing of haulm killing or harvest, the parameter values for the calculation of AUC, e.g. *A*_3_, were corrected to, respectively, the time of haulm killing or harvest. The sum of *A*_1_, *A*_2_, and *A*_3_ results in the total area under the canopy cover curve, *A*_sum_. The fitted daily *v* was used to calculate daily intercepted radiation and total dry weight production in the yield formation analysis (as explained in the “Yield Formation Analysis” section).

#### Harvests

One intermediate harvest in Cycle 1 (74–76 DAP) and two intermediate harvests each in Cycles 2 and 3 (65–86 DAP and 92–106 DAP, respectively) were performed (Table S3), each harvest on a pre-selected area of eight planted seed tubers per plot. Out of the harvested plants of the selected area, the number of main stems was counted, and the fresh weights of haulm, main stems, and tubers were assessed. Dry matter concentrations of haulm, main stems, and tubers were assessed by drying subsamples (each weighing ≥ 300 g in fresh weight) in a forced ventilated oven at 105 °C for > 48 h and calculated by dividing subsample dry weight by subsample fresh weight.

At the final harvest (139–157 DAP, Table S3), tubers of a pre-selected area of 24 planted seed tubers per plot were manually harvested. The number of established plants in this area was recorded just before canopy closure. The number and fresh weight of tubers in each size class were assessed manually in Cycle 1 and by using a phenotyping machine in Cycles 2 and 3. The size classes in Cycle 1 were 20–35, 35–40, 40–45, 45–50, 50–60, and 60 + (in mm). In Cycles 2 and 3, the size classes were 0–25, 25–28, 28–30, 30–35, 35–40, 40–45, 45–48, 48–50, 50–52, 52–55, 55–60, 60–65, 65–70, 70–75, 75–80, 80–85, and 85 + (in mm). Together, the size classes used for analysis were 0–30, 30–40, 40–45, 45–50, 50–60, 60–70, 70–80, and 80 + (in mm).

#### Yield Formation Analysis

In the yield formation analysis, *tuber yield* was analysed as being a function of the incident photosynthetically active radiation (*PAR*) during the growing period, the fraction of this radiation that was intercepted by the crop leading to the cumulative intercepted radiation (*CIR*), the radiation use efficiency (*RUE*), total dry weight (*Total DW*), harvest index (*HI*), tuber dry weight (*Tuber DW*) and tuber dry matter concentration (*Tuber DMC*).

*PAR* was calculated as 50% of the total incident global solar radiation at a field site. *CIR* was calculated over the whole growing period (from planting until haulm killing, if taken place, or final harvest) by multiplying the fitted percentage of canopy cover (*v*) and the *PAR* for every day and summing up the daily values over the entire growing period. *RUE* was calculated as the dry weight production (sum of dry weight of haulm, main stems, and tubers) at the intermediate harvest (in Cycle 1) or the second intermediate harvests (in Cycles 2 and 3), divided by the *CIR* until the respective harvest days. *Total DW* was calculated as the multiplication of *CIR* of the entire growing period and *RUE*. *Tuber yield* was calculated per m^2^ as the fresh weight of tubers per plot in the final harvest divided by the number of planted seed tubers (24 per plot) and multiplied by the planting density. To assess *Tuber DMC*, in Cycle 1, tuber under-water-weight (UWW) was assessed using > 5 kg of tubers > 50 mm (given that the difference in UWW with small tubers, 0–50 mm, was very small) and *Tuber DMC* was calculated as $0.0492\times UWW+2$ (Ludwig 1972; Veerman 2001). In Cycles 2 and 3, *Tuber DMC* was assessed by drying subsamples of mixed sizes (each subsample weighing ≥ 500 g in fresh weight) in a forced ventilated oven at 105 °C for > 48 h and calculated by dividing the subsample dry weight by the subsample fresh weight. *Tuber DW* was calculated by multiplying *Tuber yield* and *Tuber DMC*. *HI* was calculated by dividing *Tuber DW* by *Total DW*.

#### Yield Component Analysis

In the yield component analysis, *Tuber yield* was analysed as a function of earlier formed components: the *number of tubers planted per m*^*2*^ (i.e. planting density), the *fraction of harvested plants per planted seed tuber*, the number of *Main stems per plant* and *per m*^*2*^, the number of tubers (*Tuber number*) *per main stem*, *per plant*, and *per m*^*2*^, and the fresh weight per tuber (*Weight per tuber*).

The *fraction of harvested plants per planted seed tuber* was calculated as the number of harvested (established) plants in the pre-selected area for final harvest in a plot divided by the number of seed tubers planted in that area (24). The number of *Main stems per plant* was calculated by dividing the total number of main stems assessed during the intermediate harvest (in Cycle 1) or the second intermediate harvests (in Cycles 2 and 3), by the number of harvested plants out of 8 planted seed tubers in the intermediate harvest area. *Main stems per m*^*2*^ was calculated as *Main stems per plant* multiplied by the number of harvested plants per m^2^. *Tuber yield* was calculated as the fresh weight of the final harvest divided by the number of planted seed tubers (24) and further multiplied by the planting density. *Weight per tuber* was calculated as the fresh weight of the final harvest divided by the number of harvested tubers. *Tuber number per main stem* was calculated as the total number of harvested tubers divided by the number of harvested plants and further divided by the number of *Main stems per plant*. *Tuber number per plant* was calculated as *Tuber number per main stem* multiplied by the number of *Main stems per plant*. *Tuber number per m*^*2*^ was calculated as *Tuber number per main stem* multiplied by the number of *Main stems per m*^*2*^.

#### Statistical Analysis

Curve fitting of the canopy cover was performed using the non-linear least-square regression method in SAS software 9.4 (SAS Institute Inc 2018), which generated the approximate standard error and the approximate 95% confidence interval of the estimated parameter values.

Other calculations and statistical analyses using linear mixed models were performed in R Statistical Software v4.3.0. (R Core Team 2021). Due to differences in the number of sites and storage temperatures among cycles, statistical analyses were performed per cycle. Statistical analyses of linear mixed models used the ‘lme’ function in the ‘nlme’ package. The fixed effects of the linear mixed model consisted of (interactions between) storage temperature effect, cultivar effect, and site effect. The random effect of the linear mixed model consisted of a structure of the block effect nested in the site effect, as well as a selected variance structure to correct for the heterogeneity of variance. For each response variable, models with different variance structures correcting for no, single, or multiple factors of storage temperature, cultivar, and site were compared (Zuur et al. 2009). The model with the lowest Akaike information criterion (AIC) or Bayesian information criterion (BIC) was selected, and analysis of variance (ANOVA) was performed. Tukey’s HSD method was used in post hoc tests using the ‘emmeans’ package to analyse statistical differences between storage temperatures within a cycle × site × cultivar combination.

## Results

### Emergence

All treatments reached 100% emergence, except for Agria stored at 17 °C in Cycle 3 (Table 4; Fig. S7) due to the formation of ‘little potatoes’ after planting.

**Table 4:** Yield component analysis. ANOVA results of each variable are summarised per cycle with *p*-values < 0.05 in bold. Within a cycle × site (S) × cultivar (CV) combination, no overlap in letters indicates significant differences between storage temperatures (ST) in post hoc tests. No ANOVA or post hoc tests for *planted tubers per m*^*2*^ and *fraction harvested plant per planted tuber* due to the strong influence of planting distances

Cycle/S	CV	ST	Planted tubers per m^2^	Fraction harvested plant per planted seed tuber	Main stems per plant	Main stems per m^2^	Tuber number per main stem	Tuber number per plant	Tuber number per m^2^	Weight per tuber	Yield per m^2^
		°C	# / m^2^	# / #	# / plant	# / m^2^	# / stem	# / plant	# / m^2^	g / tuber	kg / m^2^
**Cycle 1**											
**Est**	**Agria**	4	4.94	1.00	2.0 a	10.1 a	3.1 ab	6.2 a	30.8 a	212 a	6.5 a
		7	4.94	1.00	1.7 a	8.2 a	4.4 b	6.1 a	30.2 a	215 a	6.5 a
		10	4.94	0.97	1.9 a	9.2 a	2.7 a	5.1 a	24.4 a	227 a	5.6 a
	**Festien**	4	4.94	1.00	3.0 a	15.0 b	2.5 a	7.5 a	36.8 a	120 a	4.4 a
		7	4.94	1.00	2.2 a	10.7 a	2.8 a	6.1 a	30.0 a	134 a	4.0 a
		10	4.94	1.00	2.5 a	12.4 a	2.7 a	6.7 a	33.1 a	131 a	4.3 a
	**Innovator**	4	4.94	1.00	3.2 a	15.8 a	2.1 a	6.7 a	32.9 a	168 a	5.5 a
		7	4.94	1.00	2.4 a	11.7 a	2.2 a	5.1 a	25.0 a	219 c	5.5 a
		10	4.94	0.99	2.3 a	11.1 a	2.7 a	6.0 a	29.4 a	189 b	5.5 a
	**Lady Claire**	4	4.94	1.00	2.8 a	13.8 a	3.5 a	9.8 a	48.2 a	94 b	4.5 a
		7	4.94	1.00	3.5 a	17.3 a	2.9 a	10.1 a	50.0 a	79 a	4.0 a
		10	4.94	1.00	3.3 a	16.5 a	3.5 a	11.5 b	56.7 b	80 a	4.5 a
**Wanroij**	**Agria**	4	4.85	0.94	2.7 a	12.4 a	3.1 a	8.3 a	38.2 a	184 b	7.0 a
		7	4.85	0.99	4.5 b	21.4 b	2.4 a	10.8 b	51.7 b	145 a	7.4 ab
		10	4.85	0.97	3.3 a	15.6 ab	3.0 a	9.6 ab	45.5 ab	185 b	8.3 b
	**Festien**	4	4.85	0.97	4.0 b	18.5 c	2.8 a	10.8 a	50.8 a	102 a	5.2 a
		7	4.85	0.86	3.7 ab	15.1 b	2.7 a	9.8 a	40.9 a	110 a	4.5 a
		10	4.85	0.90	2.9 a	12.5 a	3.5 a	10.1 a	44.0 a	108 a	4.7 a
	**Innovator**	4	4.85	0.99	4.7 b	22.6 b	1.7 a	7.8 a	37.0 a	164 a	6.1 a
		7	4.85	0.92	3.3 a	14.7 a	2.6 b	8.5 a	37.7 a	153 a	5.8 a
		10	4.85	0.90	4.7 b	21.0 ab	1.8 ab	8.3 a	36.3 a	153 a	5.5 a
	**Lady Claire**	4	4.85	0.92	4.5 a	19.8 a	3.5 a	15.4 a	68.5 a	72 a	4.9 a
		7	4.85	0.86	4.6 a	19.3 a	3.8 a	17.6 a	73.0 a	65 a	4.7 a
		10	4.85	0.92	4.0 a	17.9 a	4.0 a	15.7 a	69.6 a	68 a	4.8 a
Cycle 1 *P*-values	ST				0.190	** < 0.001**	0.263	0.841	0.755	0.745	0.410
	CV				** < 0.001**	** < 0.001**	** < 0.001**	** < 0.001**	** < 0.001**	** < 0.001**	** < 0.001**
	S				** < 0.001**	0.095	0.885	** < 0.001**	** < 0.001**	0.090	0.058
	ST × CV				** < 0.001**	** < 0.001**	**0.005**	**0.001**	** < 0.001**	** < 0.001**	0.665
	ST × S				0.278	**0.005**	0.407	**0.009**	0.128	0.143	0.624
	CV × S				0.077	**0.006**	**0.009**	**0.009**	**0.001**	** < 0.001**	**0.007**
	ST × CV × S				** < 0.001**	**0.007**	**0.020**	**0.027**	0.052	** < 0.001**	**0.013**
**Cycle 2**											
**Est**	**Agria**	4	4.76	1.00	2.8 a	13.1 a	2.6 a	7.2 a	34.1 a	139 a	4.2 a
		7	4.76	1.00	2.6 a	12.2 a	2.5 a	6.4 a	30.6 a	167 a	4.7 ab
		10	4.76	1.00	2.4 a	11.5 a	3.4 a	8.1 a	38.4 a	131 a	5.0 b
	**Festien**	4	4.76	1.00	2.6 a	12.4 a	3.3 a	8.4 a	39.8 a	95 a	3.7 a
		7	4.76	1.00	2.4 a	11.4 a	3.3 a	7.8 a	37.2 a	93 a	3.4 a
		10	4.76	1.00	2.2 a	10.4 a	3.5 a	7.5 a	35.8 a	98 a	3.4 a
	**Innovator**	4	4.76	1.00	3.1 a	14.6 a	1.9 a	5.6 a	26.5 a	176 a	4.5 a
		7	4.76	1.00	3.0 a	14.2 a	2.2 a	6.4 a	30.6 a	148 a	4.4 a
		10	4.76	1.00	3.3 a	15.6 a	1.7 a	5.5 a	26.1 a	160 a	4.0 a
	**Lady Claire**	4	4.76	1.00	4.2 a	20.1 a	1.7 a	6.8 a	32.1 a	85 b	2.7 a
		7	4.76	1.00	4.8 ab	22.8 ab	1.7 a	8.3 ab	39.6 ab	88 b	3.5 a
		10	4.76	1.00	5.3 b	25.1 b	1.9 a	9.6 b	45.6 b	71 a	3.3 a
**Nagele**	**Agria**	4	4.76	1.00	2.7 b	13.0 b	1.9 a	5.1 b	24.1 b	238 a	5.7 a
		7	4.76	1.00	2.2 ab	10.5 ab	2.3 a	5.1 b	24.5 b	220 a	5.4 a
		10	4.76	1.00	1.9 a	9.1 a	2.3 a	4.3 a	20.4 a	281 b	5.7 a
	**Festien**	4	4.76	1.00	2.5 a	12.0 a	2.0 a	5.0 a	23.7 a	159 a	3.8 a
		7	4.76	1.00	2.1 a	10.1 a	2.4 ab	5.1 a	24.3 a	144 a	3.5 a
		10	4.76	1.00	2.0 a	9.5 a	2.6 b	5.2 a	24.5 a	158 a	3.8 a
	**Innovator**	4	4.76	1.00	2.8 a	13.3 a	1.7 a	4.7 a	22.3 a	235 b	5.2 a
		7	4.76	1.00	2.7 a	12.6 a	1.9 a	4.9 a	23.4 a	210 a	4.9 a
		10	4.76	1.00	2.8 a	13.3 a	1.5 a	4.3 a	20.4 a	225 ab	4.6 a
	**Lady Claire**	4	4.76	1.00	4.9 b	23.2 b	1.6 a	7.8 a	37.1 a	115 a	4.3 a
		7	4.76	1.00	4.0 a	19.0 a	2.0 a	7.8 a	37.2 a	119 a	4.4 a
		10	4.76	1.00	4.2 a	19.8 a	1.8 a	7.6 a	36.1 a	114 a	4.1 a
**Wanroij**	**Agria**	4	4.76	1.00	3.1 a	14.8 a	2.5 a	7.7 a	36.6 a	202 b	7.4 a
		7	4.76	1.00	2.9 a	14.0 a	3.0 a	8.7 a	41.5 a	172 a	7.1 a
		10	4.76	1.00	2.7 a	12.8 a	2.9 a	7.4 a	35.4 a	199 b	7.0 a
	**Festien**	4	4.76	1.00	2.8 a	13.1 a	3.8 a	10.4 ab	49.4 ab	117 a	5.8 a
		7	4.76	1.00	2.8 a	13.1 a	4.3 a	11.5 b	55.0 b	107 a	5.9 a
		10	4.76	1.00	2.4 a	11.6 a	4.2 a	10.2 a	48.5 a	117 a	5.7 a
	**Innovator**	4	4.76	1.00	3.2 a	15.2 a	2.1 a	6.6 a	31.4 a	202 a	6.3 a
		7	4.76	1.00	3.3 a	15.5 a	2.0 a	6.6 a	31.4 a	183 a	5.7 a
		10	4.76	1.00	3.0 a	14.1 a	2.3 a	6.7 a	31.9 a	191 a	6.1 a
	**Lady Claire**	4	4.76	1.00	4.8 b	22.7 b	2.8 a	13.4 ab	63.9 ab	81 a	5.2 a
		7	4.76	1.00	4.4 ab	21.0 ab	2.8 a	12.4 a	59.3 a	81 a	4.8 a
		10	4.76	1.00	4.1 a	19.4 a	3.5 a	13.9 b	66.4 b	74 a	4.9 a
Cycle 2 *P*-values	ST				**0.002**	**0.002**	** < 0.001**	**0.033**	**0.033**	**0.016**	0.309
	CV				** < 0.001**	** < 0.001**	** < 0.001**	** < 0.001**	** < 0.001**	** < 0.001**	** < 0.001**
	S				0.200	0.201	**0.024**	**0.024**	** < 0.001**	0.272	** < 0.001**
	ST × CV				0.254	0.254	0.265	0.140	0.140	** < 0.001**	0.313
	ST × S				**0.003**	**0.003**	0.668	0.271	0.271	0.155	0.329
	CV × S				0.057	0.057	** < 0.001**	** < 0.001**	** < 0.001**	** < 0.001**	** < 0.001**
	ST × CV × S				0.066	0.066	0.856	**0.005**	**0.005**	0.468	0.153
**Cycle 3**											
**Est**	**Agria**	4	4.30	1.00	1.9 a	8.1 a	5.5 a	10.4 a	44.5 a	138 a	6.0 a
		10	4.30	1.00	2.0 a	8.4 a	3.8 a	7.4 a	31.8 a	177 a	5.3 a
		17	4.30	0.92	1.7 a	6.8 a	4.3 a	7.5 a	29.3 a	170 a	5.0 a
	**Festien**	4	4.30	1.00	1.9 a	8.2 a	4.0 a	7.6 a	32.7 a	131 a	4.3 a
		10	4.30	1.00	2.0 a	8.8 a	4.2 a	8.5 a	36.7 a	122 a	4.5 a
		17	4.30	1.00	1.9 a	8.3 a	4.0 a	7.7 a	33.2 a	133 a	4.4 a
	**Innovator**	4	4.30	1.00	1.5 a	6.5 a	3.8 b	5.8 a	24.7 a	187 a	4.6 a
		10	4.30	1.00	2.0 ab	8.7 ab	3.1 ab	6.3 a	27.0 a	173 a	4.7 a
		17	4.30	1.00	2.3 b	10.0 b	2.6 a	6.1 a	26.1 a	173 a	4.5 a
	**Lady Claire**	4	4.30	1.00	2.8 a	11.9 a	4.2 a	10.7 a	46.0 a	102 a	4.4 a
		10	4.30	1.00	3.2 a	13.7 a	2.7 a	8.6 a	37.2 a	112 a	3.7 a
		17	4.30	1.00	3.8 b	16.5 b	2.5 a	9.8 a	42.1 a	85 a	3.4 a
**Nagele**	**Agria**	4	4.76	1.00	2.5 b	11.9 b	3.7 a	9.1 b	43.3 b	135 a	5.8 a
		10	4.76	1.00	3.0 b	14.5 c	3.2 a	9.5 b	45.4 b	130 a	5.9 a
		17	4.76	0.81	1.8 a	6.8 a	4.2 a	7.4 a	28.4 a	184 b	5.2 a
	**Festien**	4	4.76	1.00	2.7 a	12.8 a	3.1 a	8.3 a	39.3 a	115 a	4.5 b
		10	4.76	1.00	3.0 a	14.1 a	3.0 a	8.8 a	41.9 a	105 a	4.4 ab
		17	4.76	1.00	2.9 a	13.8 a	3.0 a	8.4 a	40.0 a	103 a	4.1 a
	**Innovator**	4	4.76	1.00	1.6 a	7.6 a	3.8 a	5.9 ab	28.0 ab	186 b	5.2 a
		10	4.76	1.00	1.9 a	9.2 a	3.6 a	6.8 b	32.5 b	154 a	5.0 a
		17	4.76	0.97	1.8 a	8.4 a	3.2 a	5.8 a	26.7 a	183 ab	4.8 a
	**Lady Claire**	4	4.76	1.00	2.9 a	14.0 a	4.2 a	12.3 a	58.5 a	80 b	4.7 b
		10	4.76	1.00	3.5 b	16.8 b	4.2 a	14.6 c	69.6 c	68 a	4.7 b
		17	4.76	1.00	3.4 ab	16.4 ab	3.9 a	13.4 b	64.0 b	66 a	4.2 a
**Wanroij**	**Agria**	4	4.30	0.99	2.7 ab	11.2 b	3.4 a	9.0 a	38.3 a	137 a	5.2 a
		10	4.30	0.99	3.1 b	13.1 b	3.2 a	9.8 a	41.5 a	136 a	5.6 a
		17	4.30	0.89	2.2 a	8.3 a	3.7 a	7.9 a	30.5 a	188 b	5.6 a
	**Festien**	4	4.30	1.00	2.8 a	12.1 a	3.2 a	9.0 a	38.7 a	104 a	4.0 a
		10	4.30	1.00	2.9 a	12.6 a	3.4 a	9.8 a	42.1 a	96 a	4.0 a
		17	4.30	1.00	3.1 a	13.3 a	3.1 a	9.5 a	40.8 a	97 a	3.9 a
	**Innovator**	4	4.30	1.00	2.0 a	8.7 a	2.6 a	5.2 ab	22.3 ab	186 a	4.1 b
		10	4.30	1.00	2.0 a	8.6 a	2.9 a	5.7 b	24.4 b	175 a	4.3 b
		17	4.30	1.00	1.9 a	8.3 a	2.6 a	4.9 a	21.0 a	145 a	3.0 a
	**Lady Claire**	4	4.30	1.00	3.8 a	16.1 a	3.1 a	11.7 a	50.4 a	92 a	4.6 a
		10	4.30	1.00	3.8 a	16.4 a	3.3 a	12.5 a	53.7 a	89 a	4.8 a
		17	4.30	0.99	3.7 a	15.8 a	3.2 a	12.1 a	51.1 a	85 a	4.3 a
Cycle 3 *P*-values	ST				** < 0.001**	** < 0.001**	0.450	** < 0.001**	** < 0.001**	** < 0.001**	** < 0.001**
	CV				** < 0.001**	** < 0.001**	** < 0.001**	** < 0.001**	** < 0.001**	** < 0.001**	** < 0.001**
	S				**0.016**	**0.008**	0.528	0.915	0.537	0.599	0.802
	ST × CV				** < 0.001**	** < 0.001**	0.066	** < 0.001**	** < 0.001**	** < 0.001**	0.544
	ST × S				**0.003**	**0.003**	0.172	0.316	0.086	0.425	0.258
	CV × S				** < 0.001**	** < 0.001**	** < 0.001**	** < 0.001**	** < 0.001**	** < 0.001**	** < 0.001**
	ST × CV × S				0.206	0.076	0.699	0.663	0.460	0.268	0.057

**Fig. 1:**
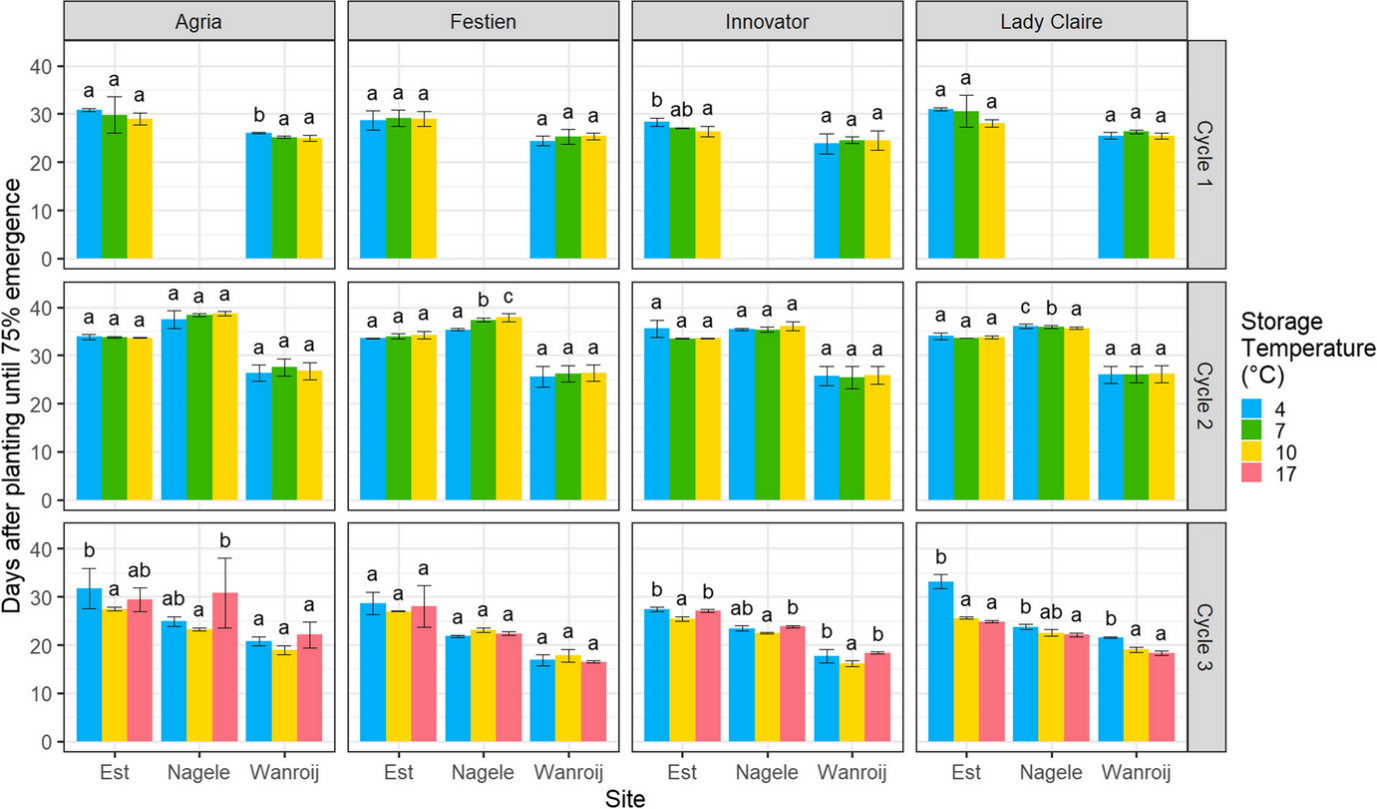
Days after planting until 75% emergence was reached of planted seed tubers of four cultivars stored at different temperatures (in colours) at three sites in three cycles. Error bars indicate ± standard deviation based on three blocks. No overlap in letters indicates a significant difference between storage temperatures within a cycle × site × cultivar combination in post hoc tests

Seed tubers of Agria and Innovator stored at 4 °C and 17 °C exhibited delayed emergence (longer time to reach 75% emergence) particularly in Cycle 3 (Fig. 1). Seed tubers of Lady Claire stored at higher storage temperatures (10 °C and 17 °C) exhibited accelerated emergence (shorter time to reach 75% emergence) particularly in Cycle 3, while Festien stored at lower storage temperatures exhibited accelerated emergence in Cycle 2 at one site (Nagele) (Fig. 1).

### Fitted Canopy Cover Curve

**Fig. 2:**
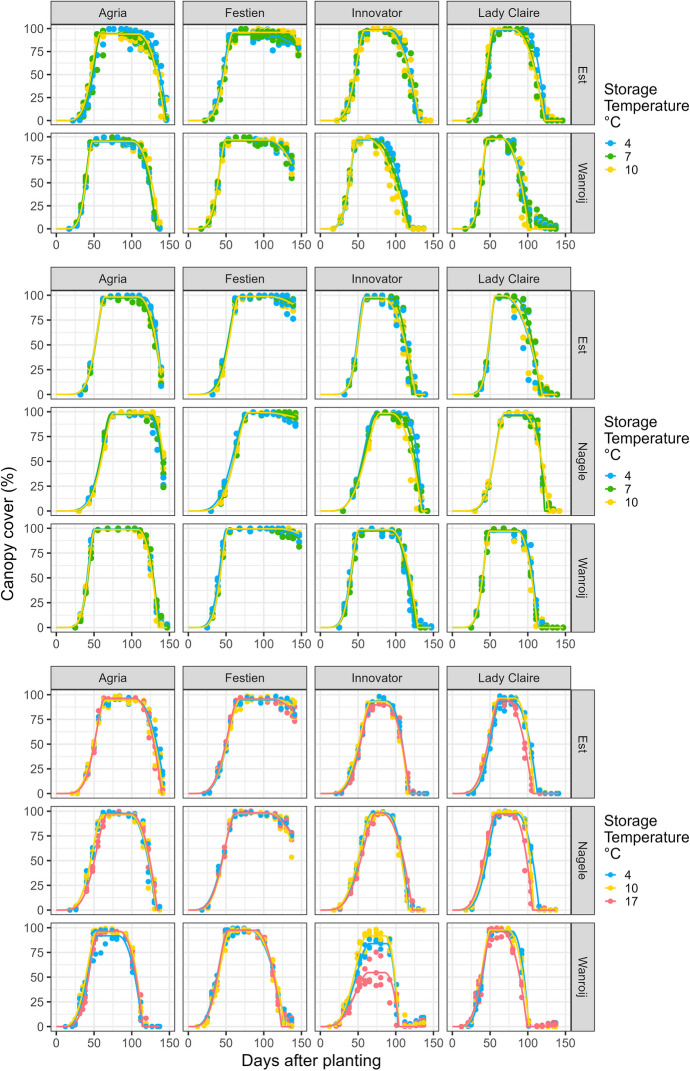
Development of canopy cover (%) from seed tubers of four cultivars stored at different temperatures (in colours) at three sites in Cycles 1 to 3 (from top to bottom). Lines are fitted curves, points are observations (three blocks), and accompanying parameter values are listed in Table S4

Nearly all crops achieved canopy closure (Fig. 2), with fitted values of maximum canopy cover (*v*_max_) mostly above 95% and approaching 100% (Table S4). Overall, few and small differences were observed between storage temperatures in the fitted canopy cover curve in each of the three phases: the build-up phase *P*1, the maximum canopy cover phase *P*2, and the canopy decline phase *P*3 (Fig. 2; Table S4). Statistically significant differences are described below per cultivar. Notably, Innovator stored at 17 °C did not reach canopy closure in Cycle 3 at one of the sites (Wanroij), and Lady Claire stored at 17 °C demonstrated consistent early onsets of senescence in Cycle 3 across sites. Details of fitted parameter values, standard errors, and calculated AUCs are listed in Table S4.

For Agria, seed tubers stored at 17 °C showed slower and delayed canopy build-up in Cycle 3, illustrated by a longer time to reach the inflexion point in *P*1 (increased *t*_m1_) at two sites (Nagele and Wanroij) and the end of *P*1 (increased *t*_1_) at one site (Nagele), compared to when stored at lower temperatures (Fig. 2; Table S4). Note that in Cycle 2 at two sites (Est and Nagele), Agria did not reach complete senescence by the time of haulm killing (Fig. 2; Table S3). Agria stored at 17 °C showed earlier full senescence than when stored at 4 °C in Cycle 3 at one site (Est), illustrated by a shorter time to reach the end of *P*3 (reduced *t*_e_).

For Festien, seed tubers stored at 4 °C showed faster canopy build-up (reduced *t*_m1_) compared to when stored at higher temperatures. Seed tubers stored at 17 °C showed an earlier onset of senescence than when stored at 4 °C in Cycle 3 at one site (Est), illustrated by a shorter time to reach the end of *P*2 (reduced *t*_2_). Seed tubers stored at 17 °C showed earlier full senescence (reduced *t*_e_) in Cycle 3 at one site (Wanroij) than tubers stored at lower temperatures. In most other cases, Festien was still in the early phase of canopy decline (*P*3) at the end of the experimental period, with the final canopy cover being mostly above 75% (Fig. 2; Table S4).

For Innovator, seed tubers stored at 17 °C showed slower canopy build-up (increased *t*_m1_ and *t*_1_) compared to when stored at lower temperatures in Cycle 3 at two sites (Est and Nagele). Lower storage temperatures delayed the onset of senescence (increased *t*_2_) compared to higher storage temperatures in a few sites and cycles. Seed tubers stored at 17 °C showed delayed full senescence (increased *t*_e_) compared to those stored at lower temperatures in Cycle 3 at two sites (Est and Nagele). Meanwhile, seed tubers stored at 17 °C failed to achieve canopy closure (reduced *v*_max_) in Cycle 3 at one site (Wanroij), displaying severely stunted growth (Fig. 2; Table S4).

For Lady Claire, higher storage temperatures induced faster initial canopy build-up (reduced *t*_m1_) in a few cases, and in Cycle 3, they generally advanced the onset of senescence (reduced *t*_2_) and full senescence (reduced *t*_e_) compared to lower storage temperatures (Fig. 2; Table S4).

### Yield Analysis

No clear or consistent effects of storage temperature on final tuber yield were observed (Table 4; Fig. S8). The lack of large differences in final yield among storage temperatures was in line with the overall lack of large differences in the yield-determining variables. These covered variables in the yield formation analysis, including the cumulative intercepted radiation (*CIR*), radiation use efficiency (*RUE*), total dry weight production (*Total DW*), harvest index (*HI*), tuber dry weight (*Tuber DW*) and tuber dry matter concentration (*Tuber DMC*) (Tables S4 and S5) and variables in the yield components analysis, including the number of main stems (per plant or per m^2^), number of tubers (per main stem, per plant, or per m^2^) and average tuber weight (*Weight per tuber*) (Table 4). Statistically significant differences are presented below per cultivar, among which Innovator stored at 17 °C showed severe yield reduction in Cycle 3 at one site (Wanroij).

For Agria, seed tubers stored at 4 °C yielded 16% less than when stored at 10 °C in Cycle 1 at one site (Wanroij) (Table 4; Fig. S8), which was associated with fewer established plants (Table 4), earlier full senescence (increased *t*_e_, Table S4) and thus lower AUC in *P*1 (*A*_1_) and *P*3 (*A*_3_) and lower total AUC (*A*_sum_) (Fig. 2; Table S4). Seed tubers stored at 4 °C also yielded 16% less than when stored at 10 °C in Cycle 2 at one site (Est) (Table 4; Fig. S8), which was probably associated with the later achievement of maximum soil cover (*t*_1_), lower harvest index (0.62 vs 0.80 g g^−1^) and lower tuber yield in dry weight (973 vs 1149 g m^−2^), although not statistically significant (Tables 4 and S4). Despite not resulting in differences in yield, Agria seed tubers stored at 17 °C in Cycle 3 established around 10% fewer plants in the final harvest area at all three sites and produced fewer main stems (per plant and per m^2^) and fewer (per plant and per m^2^) yet heavier (Table 4) and larger (Fig. S9) tubers than when stored at lower temperatures.

For Festien, seed tubers stored at 17 °C yielded 9% less than when stored at 4 °C in Cycle 3 at one site (Nagele) without clear attributable differences in the analysis components. Seed tubers stored at 17 °C led to lower *Tuber DMC* than when stored at 10 °C in Cycle 3 at one site (Wanroij). In general, very few and limited differences between storage temperatures were found for Festien, which is probably linked to the fact that Festien had not completed senescence by the end of the experimental periods.

For Innovator, seed tubers stored at 17 °C yielded around 30% less than when stored at lower storage temperatures in Cycle 3 at one site (Wanroij) (Table 4; Fig. S8), which could be attributed to a lower total AUC (*A*_sum_) (Fig. 2; Table S4), lower cumulative intercepted radiation (Table S4), and lower total and tuber dry weight production (*Total DW*) (Table S4). Seed tubers stored at 17 °C produced more stems (per plant and per m^2^) in Cycle 3 at one site (Est) and produced fewer tubers (per stem, per plant, or per m^2^) at three sites than when stored at lower temperatures (Table 4).

For Lady Claire, seed tubers stored at 17 °C yielded around 11% less than when stored at lower storage temperatures in Cycle 3 at one site (Nagele) (Table 4; Fig. S8), which was associated with a lower total AUC (*A*_sum_) (Fig. 2; Table S4) and lower total dry weight production (*Total DW*) (Table S4). Seed tubers stored at 17 °C in Cycle 3 produced more stems (per plant and per m^2^) at two sites (Est and Nagele) and produced tubers with lower tuber dry matter concentration (*Tuber DMC*) at two sites (Nagele and Wanroij) than tubers stored at lower temperatures (Table S5).

## Discussion

### General Lack of Storage Temperature Effects on Crop Performance

Based on the sprouting behaviour in sprouting tests during storage (Fig. S1, Zou et al. 2024), we assumed that by the time of planting, a storage temperature of 17 °C would lead to physiologically old seed tubers across all cultivars, 10 °C would create physiologically rather old seed tubers in Festien and Innovator, 4 °C would produce physiologically very young seed tubers in Agria and Lady Claire, while the remaining cultivar-storage temperature combinations would result in seed tubers around the optimal physiological age. Our hypothesis postulated that optimally aged seed tubers would demonstrate robust growth vigour in the field and high-yielding capacity, whereas those physiologically too young or too old would exhibit lower growth vigour and higher sensitivity to suboptimal growth conditions (weather and soil), resulting in poorer crop performance.

Contrary to our hypothesis, temperature-induced differences in sprouting behaviour did not translate into consistent or significant variations in field performance across cycles and sites (Figs. 1 and 2; Tables 4 and S5). Mostly minor differences in emergence, canopy cover development, stem and tuber number, and other yield-related attributes led to small or absent yield differences (Fig. 2; Tables 4 and S4). In particular, no clear and conclusive differences were observed among storage temperatures in the range of 4 to 10 °C, despite more than a twofold increase in the storage T-sum within this interval (Table 2). In a previous study, Reust et al. (2001) reported up to a 50% yield loss of Agria for seed tubers stored until a T-sum of 4400 °C, accumulated from the tuber set to the planting of the seed tuber. However, such strong yield depression was not observed for Agria nor any other cultivar in the present study, even with the corresponding T-sums exceeding 4400 °C (Table 2).

The absence of clear differences could be assumed resulting from several aspects. First, despite the observed differences in sprouting behaviour after storage at 4 to 10 °C, seed tubers may still have been in the phase of optimal growth vigour at planting, retaining good resilience to adapt to field environments. Nutrient quantity tests at planting in Cycle 3 revealed similar nutrient contents in seed tubers stored at 10 °C compared with 4 °C, except for lower nitrogen levels (Table S1). To observe the effect of physiological age on field performance, it might be necessary to further diversify growth vigour, such as incorporating a wider range of storage temperatures and corresponding total T-sums or changing patterns of building up storage T-sums, as demonstrated in prior studies (Gillison et al. 1987; Scholte 1987; Van Ittersum and Scholte 1992; Struik et al. 2006).

Second, differences in physiological age may only manifest under extreme environmental conditions. Despite different weather conditions, such as a dry spring with a late heat wave in Cycle 1 (2020), a prolonged wet spring in Cycle 2 (2021), and a dry hot summer in Cycle 3 (2022), average temperatures during the growing seasons remained relatively stable at around 16 to 17 °C (Table 3; Figs. S3 and S4), which are optimal for potato plants growth (Struik 2007).

Furthermore, proper field management, including fertiliser applications (Table S2), irrigation at two sites (Est and Wanroij) (Table S2), and weekly disease control, might have suppressed the expression of physiological age. It is known that nitrogen fertilisation extends the duration of haulm growth and increases yield if seasons are long (Struik and Wiersema 1999), potentially prolonging the life cycle of crops grown from physiologically old seed tubers. In addition, the careful crop management by the participating farmers might have mitigated a negative impact of occasional harsh environmental events on crop performance.

Finally, a different hypothesis contrary to the previous assumptions is that the effects of physiological age only emerge under optimal growth conditions. In less favourable growth conditions, the effects may be largely influenced or overruled by these environmental conditions. Wurr (1978) reported a large effect of storage temperature on yield in 1973 when the conditions for growth were optimal, whereas the effects were small in subsequent years with hot dry weather. Previous studies also reported a high yearly variation of storage temperature effect on yield, as well as differences in crop performance between controlled and field conditions (Wurr 1978; Van der Zaag and Van Loon 1987; Hay and Hampson 1991). In this study, observations of reduced yield due to water excess in clayey soils in Cycle 2 and drought stress in the sandy soil in Cycle 3 (Figs. S4 and S8) align with recent findings on the potato yield gap in the Netherlands by Ravensbergen et al. (2024). Within a studied cultivar, in most cases cycle and site contributed to larger yield differences than storage temperature (Table 4; Fig. S8).

### Some Differences in Early Growth Vigour Caused by Storage Temperature

Despite the general absence of strong effects, significant effects of storage temperature were observed in some cycle × site × cultivar combinations during the early stages of crop development, including emergence and canopy build-up (Figs. 1 and 2; Table S4). Cultivars subjected to storage temperatures inducing accelerated or decelerated emergence also exhibited, respectively, accelerated or decelerated canopy build-up. This was evident in Agria and Innovator stored at 17 °C, Festien at lower temperatures, and Lady Claire at higher temperatures. During later phases, however, the effects of storage at 4 to 10 °C were usually inconsistent across sites. The finding that physiological age has more pronounced influences on the early stages of plant growth compared to the late stages aligns with previous findings (Perennec and Madec 1980; Van der Zaag and Van Loon 1987; Benz and Fahem 1988; Knowles and Botar 1991; Van Ittersum 1993), indicating that early crop performance is more prominently governed by the inherent properties of the seed tubers, while the impact of environmental conditions becomes more predominant as the growing season progresses.

### Some Differences Caused by Storage at 17 °C

In addition to influencing early growth stages, significant effects of storage temperature on field performance were observed in some site × cultivar combinations when a storage temperature of 17 °C was included in Cycle 3. Table 5 summarises the observed effects of storage at 17 °C across the three sites.

**Table 5:** Occurrence of significant effects of storage at 17 °C compared to lower temperatures (i.e. 4 and/or 10 °C) on the field performance in Cycle 3 at three sites. ‘ + ’ or ‘ − ’ indicates significant differences found at one site, ‘ +  + ’ or ‘––’ at two sites, ‘ +  +  + ’ or ‘ −  −  − ’ at three sites, and ‘0’ indicates significant differences not found

Cultivar	Rate of ageing	Maturity	Emergence		Canopy				Main stem	Tuber			
			Speed^1^	Final number^2^	Build-up^1^	Max.^2^	Onset senescence^3^	Full senescence^3^	Number^2^	Number^2^	Yield^2^	Size^2^	DMC^2^
Agria	Slow	Late	−	− − −	− −	0	0	+	− −	−	0	+ +	0
Festien	Fast	Late	0	0	0	0	+	+	0	0	−	0	−
Innovator	Fast	Early	− − −	0	− −	−	+	− −	+	− −	−	0	0
Lady Claire	Slow	Early	+ + +	0	+ +	0	+ + +	+ +	+ +	0	−	−	−

*DMC* dry matter concentration

^1^ + faster, − slower

^2^ + higher, − lower

^3^ + advanced, − delayed

Despite that a storage temperature of 17 °C created physiologically old seed tubers in all cultivars in Cycle 3 (Fig. S1; Zou et al. 2024), effects on the field performance variables across growth stages were specific to cultivars (Table 5): for Agria (slow ageing and late maturity), storage at 17 °C resulted in slower emergence, reduced final emergence, slower canopy build-up, advanced full senescence, fewer main stems (per plant and m^2^) and tubers (per plant and m^2^) and increased tuber size, at some or all sites, compared to one or both of the lower storage temperatures; for Festien (fast ageing and late maturity), storage at 17 °C resulted in advanced senescence, reduced yield and reduced tuber dry matter concentration, at some sites, compared to one or both of the lower storage temperatures; for Innovator (fast ageing and early maturity), storage at 17 °C resulted in later emergence, slower canopy build-up, reduced maximum canopy cover, advanced onset of senescence, delayed full senescence, more main stems (per plant and m^2^), fewer tubers (per plant and m^2^), and reduced yield, at some or all sites, compared to one or both of the lower storage temperatures; and for Lady Claire (slow ageing and early maturity), storage at 17 °C resulted in faster emergence, earlier initial canopy build-up, advanced onset of senescence and full senescence, more main stems (per plant and m^2^), reduced yield, reduced tuber size and reduced tuber dry matter concentration, at some or all sites, compared to one or both of the lower storage temperatures.

As illustrated above, the effect of 17 °C storage was highly cultivar-specific. Few consistent patterns were observed across cultivars with similar rates of ageing (Agria and Lady Claire; Festien and Innovator) or maturity types (Agria and Festien; Innovator and Lady Claire) (Table 5). Within a cultivar, the effects of 17 °C storage were mostly consistent across sites, as demonstrated by the absence of contradictory signs (‘ + ’ and ‘ − ’) coexisting within a variable (Table 5).

A storage temperature of 17 °C generally reduced tuber yield and quality, with the most severe yield depression observed for Innovator in Cycle 3 at Wanroij. One plausible explanation for this strong effect could be attributed to herbicide application. Among plants displaying symptoms of yellow-coloured leaves, Innovator suffered the most, with seed tubers stored at 17 °C exhibiting stunted growth, reduction of canopy cover up to 50% (Fig. 2; Table S4), and yield loss up to 30% (Table 4; Fig. S8). For cultivars sensitive to herbicides and other environmental stresses, physiologically old seed tubers might increase susceptibility to damage and decrease overall resilience.

## Practical Implications

In conclusion, the influence of the constant storage temperature treatments in the range of 4 to 10 °C on subsequent crop performance was small, and, if apparent, inconsistent across sites and cycles. Seed tubers stored within this temperature range generally exhibited optimal growth vigour, suggesting that stringent cold storage regimes may not be necessary for achieving proper crop performance and yield for the studied cultivars in the Netherlands. The finding that cold storage is not essential for good seed performance aligns with studies conducted in other regions, including Sri Lanka (Carls and Caesar 1979), Peru (Wiersema and Booth 1985), Tunisia (Benz and Fahem 1988), and New Zealand (Oliveira et al. 2017), although other studies have reported beneficial effects of low storage temperature (Iritani et al. 1983; Van der Zaag and Van Loon 1987). Conversely, a storage temperature of 17 °C more often resulted in reduced yield in certain cultivars and sites, indicating that such high storage temperatures should be avoided in regions with a single growing season per year and long storage periods.

## Supplementary Information

Below is the link to the electronic supplementary material.

Supplementary file1 (DOCX 7424 KB)

## Data Availability

Data can be made available upon reasonable request to the corresponding author.
