# Supplementary material for: Physiological Age of Potato Seed Tubers of Contrasting Cultivars Hardly Affects Crop Performance in a Temperate Climate
Source: Potato Res. 2024 May 20;68(1):187–217. doi: 10.1007/s11540-024-09731-2 (PMC11982108; doi:10.1007/s11540-024-09731-2)
Supplement: Supplementary file 1 — Supplementary file1 (DOCX 7424 KB) [file 11540_2024_9731_MOESM1_ESM.docx]

**Physiological age of potato seed tubers of contrasting cultivars hardly affects crop performance in a temperate climate**

Chunmei Zou^1,2^, Peter E. L. van der Putten^1^, Marieke Datema^2,3^, Leon Mossink^1^, Willemien J. M. Lommen^1^, Paul C. Struik^1^, Martin K. van Ittersum^2^

^1^ Centre for Crop Systems Analysis, Wageningen University and Research, P.O. Box 430, 6700 AK Wageningen, The Netherlands

^2^ Plant Production Systems, Wageningen University and Research, P.O. Box 430, 6700 AK Wageningen, The Netherlands

^3^ Present address: Solynta, 6703 HA Wageningen, The Netherlands

Correspondence e-mail: chunmei.zou@wur.nl

**Supplementary Information**

Tables and Figures

**Table S1**. Nutrient quantity (in mg) (with s.e. in parentheses) per de-sprouted seed tuber in Cycle 3 at planting time (May 2022). Based on ANOVA and post-hoc tests, *p*-values < 0.05 are in bold.

| **CV/ST** |  | **N** | **P** | **K** | **Ca** | **Mg** | **Fe** | **Zn** | **S** | **Mn** | **Al** |
| --- | --- | --- | --- | --- | --- | --- | --- | --- | --- | --- | --- |
| Agria |  |  |  |  |  |  |  |  |  |  |  |
| 4 |  | 131.0 (3.4) | 17.3 (1.7) | 182.2 (5.9) | 12.3 (3.3) | 7.8 (0.5) | 0.92 (0.09) | 0.12 (0.01) | 12.1 (0.8) | 0.05 (0.01) | 0.77 (0.12) |
| 10 |  | 110.8 (2.3) | 16.2 (0.4) | 168.6 (2.9) | 8.3 (0.2) | 6.8 (0.1) | 0.79 (0.00) | 0.10 (0.00) | 10.8 (0.1) | 0.04 (0.00) | 0.65 (0.09) |
| 17 |  | 102.8 (16.1) | 15.3 (2.2) | 156.7 (27.7) | 7.6 (1.5) | 6.0 (1.1) | 0.86 (0.16) | 0.08 (0.01) | 10.1 (1.5) | 0.04 (0.01) | 0.67 (0.07) |
| Festien |  |  |  |  |  |  |  |  |  |  |  |
| 4 |  | 119.0 (6.7) | 16.6 (0.7) | 176.3 (3.1) | 6.9 (0.6) | 7.4 (0.2) | 0.63 (0.03) | 0.12 (0.00) | 10.1 (0.3) | 0.04 (0.00) | 0.46 (0.04) |
| 10 |  | 88.8 (7.6) | 12.3 (0.4) | 147.0 (10.4) | 5.9 (0.3) | 5.9 (0.3) | 0.60 (0.05) | 0.09 (0.01) | 7.8 (0.4) | 0.03 (0.00) | 0.54 (0.10) |
| 17 |  | 92.5 (6.0) | 13.4 (1.7) | 156.9 (13.5) | 6.4 (0.2) | 6.0 (0.6) | 0.59 (0.03) | 0.09 (0.01) | 8.3 (0.9) | 0.03 (0.00) | 0.48 (0.03) |
| Innovator |  |  |  |  |  |  |  |  |  |  |  |
| 4 |  | 116.1 (7.0) | 13.4 (0.9) | 143.4 (4.1) | 9.2 (0.3) | 7.5 (0.3) | 0.80 (0.04) | 0.10 (0.01) | 12.1 (0.7) | 0.04 (0.00) | 0.64 (0.10) |
| 10 |  | 105.7 (6.3) | 11.0 (0.5) | 132.0 (11.8) | 8.1 (0.6) | 7.1 (0.6) | 0.77 (0.08) | 0.09 (0.01) | 11.1 (0.9) | 0.03 (0.01) | 0.65 (0.11) |
| 17 |  | 75.3 (5.8) | 7.8 (0.3) | 101.2 (8.6) | 6.0 (0.5) | 4.8 (0.4) | 0.74 (0.09) | 0.06 (0.00) | 8.1 (0.6) | 0.03 (0.01) | 0.67 (0.15) |
| Lady Claire | |  |  |  |  |  |  |  |  |  |  |
| 4 |  | 89.4 (9.0) | 14.5 (1.2) | 134.1 (5.1) | 4.6 (0.2) | 6.3 (0.6) | 0.61 (0.04) | 0.08 (0.01) | 8.0 (0.8) | 0.03 (0.00) | 0.47 (0.05) |
| 10 |  | 90.6 (5.0) | 14.3 (0.7) | 134.0 (9.9) | 4.8 (0.1) | 6.1 (0.5) | 0.68 (0.05) | 0.08 (0.01) | 8.5 (0.8) | 0.04 (0.00) | 0.53 (0.10) |
| 17 |  | 77.0 (9.4) | 12.5 (1.7) | 113.9 (10.8) | 4.2 (0.1) | 4.6 (0.4) | 0.65 (0.02) | 0.07 (0.01) | 6.3 (0.7) | 0.03 (0.00) | 0.52 (0.03) |
| *p*-values |  |  |  |  |  |  |  |  |  |  |  |
| CV |  | **0.002** | **< 0.001** | **< 0.001** | **< 0.001** | 0.083 | **< 0.001** | **0.003** | **< 0.001** | **0.011** | **0.003** |
| ST |  | **< 0.001** | **0.003** | **0.013** | **0.032** | **< 0.001** | 0.748 | **< 0.001** | **0.002** | **0.014** | 0.992 |
| CV×ST |  | 0.272 | 0.342 | 0.663 | 0.337 | 0.465 | 0.865 | 0.430 | 0.284 | 0.818 | 0.858 |
| Contrast |  |  |  |  |  |  |  |  |  |  |  |
| 4 ̶ 10 |  | 14.9 | 2.0 | 13.6 | 1.5 | 0.8 | 0.03 | 0.01 | 1.01 | 0.00 | 0.00 |
| 4 ̶ 17 |  | 27.0 | 3.2 | 26.9 | 2.2 | 1.9 | 0.03 | 0.03 | 2.35 | 0.01 | 0.00 |
| 10 ̶ 17 |  | 12.1 | 1.2 | 13.3 | 0.7 | 1.1 | 0.00 | 0.01 | 1.34 | 0.00 | 0.01 |
| *p*-values |  |  |  |  |  |  |  |  |  |  |  |
| 4 ̶ 10 |  | **0.043** | 0.057 | 0.216 | 0.180 | 0.103 | 0.766 | 0.073 | 0.216 | 0.138 | 0.996 |
| 4 ̶ 17 |  | **< 0.001** | **0.002** | **0.006** | **0.029** | **< 0.001** | 0.752 | **< 0.001** | **0.001** | **0.006** | 0.999 |
| 10 ̶ 17 |  | 0.115 | 0.332 | 0.233 | 0.645 | **0.019** | 1.000 | **0.029** | 0.074 | 0.338 | 0.990 |

**Table S2** Fertilisation and irrigation at three sites in three cycles

| **Site** | **Cycle** |  | **Fertiliser** | | | | |  | **Irrigation** | |
| --- | --- | --- | --- | --- | --- | --- | --- | --- | --- | --- |
|  |  |  | Name | Amount | N content | P content | K content |  | Events | Total |
|  |  |  |  | ha^-1^ | ton^-1^ | ton^-1^ | ton^-1^ |  | # | mm ha^-1^ |
| **Est** | **Cycle 1** |  | Mushroom compost | 50 m^3^ | 6.90 kg | 1.79 kg | 7.22 kg |  | 4 | 80 |
|  |  |  | KAS | 900 kg | 27% |  |  |  |  |  |
|  |  |  | KALI60 | 250 kg |  |  | 50% |  |  |  |
|  | **Cycle 2** |  | Mushroom compost | 80 m^3^ | 6.90 kg | 1.79 kg | 7.22 kg |  | 0 | 0 |
|  |  |  | Slurry | 25 m^3^ | 4.68 kg | 1.06 kg | 4.57 kg |  |  |  |
|  |  |  | KAS | 550 kg | 27% |  |  |  |  |  |
|  |  |  | KALI60 | 250 kg |  |  | 50% |  |  |  |
|  |  |  | Urea | 52 kg | 39% |  |  |  |  |  |
|  | **Cycle 3** |  | Mushroom compost | 60 m^3^ | 6.90 kg | 1.79 kg | 7.22 kg |  | 4 | 80 |
|  |  |  | Slurry | 28 m^3^ | 4.68 kg | 1.06 kg | 4.57 kg |  |  |  |
|  |  |  | KAS | 700 kg | 27% |  |  |  |  |  |
|  |  |  | KALI60 | 150 kg |  |  | 50% |  |  |  |
|  |  |  | Urea | 20 kg | 39% |  |  |  |  |  |
| **Nagele** | **Cycle 2** |  | Slurry | 40 m^3^ | 4.0 kg | 1.4 kg | 4.5 kg |  | 0 | 0 |
|  |  |  | KAS | 700 kg | 27% |  |  |  |  |  |
|  |  |  | Protamylasse | 300 kg | 2% |  | 7% |  |  |  |
|  | **Cycle 3** |  | Slurry | 40 m^3^ | 4.0 kg | 1.4 kg | 4.5 kg |  | 0 | 0 |
|  |  |  | KAS | 800 kg | 27% |  |  |  |  |  |
|  |  |  | Protamylasse | 300 kg | 2% |  | 7% |  |  |  |
| **Wanroij** | **Cycle 1** |  | Slurry | 28 m^3^ | 4.00 kg | 0.65 kg | 4.57 kg |  |  |  |
|  |  |  | KAS | 325 kg | 27% |  |  |  | 0 | 0 |
|  |  |  | PatentKali | 200 kg |  |  | 25% |  |  |  |
|  | **Cycle 2** |  | Slurry | 28 m^3^ | 4.00 kg | 0.65 kg | 4.57 kg |  | 5 | 125 |
|  | **Cycle 3** |  | Slurry | 28 m^3^ | 4.00 kg | 0.65 kg | 4.57 kg |  | 3 | 75 |
|  |  |  | KAS | 150 kg | 27% |  |  |  |  |  |

**Table S3** Dates and days after planting (DAP) of field activities

| **Cycle** |  | **Planting** |  |  | **Intermediate harvest 1** | |  | **Intermediate harvest 2** | |  | **Haulm killing** | |  | **Final harvest** | |
| --- | --- | --- | --- | --- | --- | --- | --- | --- | --- | --- | --- | --- | --- | --- | --- |
| **/ Site** |  | Date | DAP |  | Date | DAP |  | Date | DAP |  | Date | DAP |  | Date | DAP |
| **Cycle 1** |  |  |  |  |  |  |  |  |  |  |  |  |  |  |  |
| **Est** |  | 28-Apr-20 | 0 |  | 13-Jul-20 | 76 |  | ̶ | ̶ |  | ̶ | ̶ |  | 23-Sep-20 | 148 |
| **Wanroij** |  | 7-May-20 | 0 |  | 20-Jul-20 | 74 |  | ̶ | ̶ |  | ̶ | ̶ |  | 2-Oct-20 | 148 |
| **Cycle 2** |  |  |  |  |  |  |  |  |  |  |  |  |  |  |  |
| **Est** |  | 1-May-21 | 0 |  | 22-Jul-21 | 82 |  | 11-Aug-21 | 102 |  | 20-Sep-21 | 142 |  | 29-Sep-21 | 151 |
| **Nagele** |  | 28-Apr-21 | 0 |  | 23-Jul-21 | 86 |  | 12-Aug-21 | 106 |  | 18-Sep-21 | 143 |  | 24-Sep-21 | 149 |
| **Wanroij** |  | 7-May-21 | 0 |  | 28-Jul-21 | 82 |  | 10-Aug-21 | 95 |  | ̶ | ̶ |  | 5-Oct-21 | 151 |
| **Cycle 3** |  |  |  |  |  |  |  |  |  |  |  |  |  |  |  |
| **Est** |  | 5-May-22 | 0 |  | 13-Jul-22 | 69 |  | 9-Aug-22 | 96 |  | ̶ |  |  | 30-Sep-22 | 148 |
| **Nagele** |  | 6-May-22 | 0 |  | 15-Jul-22 | 70 |  | 10-Aug-22 | 96 |  | ̶ |  |  | 22-Sep-22 | 139 |
| **Wanroij** |  | 9-May-22 | 0 |  | 13-Jul-22 | 65 |  | 9-Aug-22 | 92 |  | ̶ |  |  | 13-Oct-22 | 157 |

**Table** **S4** Left: estimated parameter values (with s.e. of the estimates in parentheses) of the fitted function for the development of canopy cover after planting. *t*_m1_ ̶ days after planting until the inflexion point in the build-up phase *P*1, *t*_1_ ̶ days after planting until the end of *P*1, *t*_2_ ̶ days after planting until the end of the maximum canopy cover phase *P*2, *t*_e_ ̶ days after planting until the end of the canopy decline phase *P*3, *v*_max_ ̶ maximum canopy cover, *A*_1_ ̶ area under the curve in *P*1, *A*_2_ ̶ area under the curve in *P*2, *A*_3_ ̶ area under the curve in *P*3, *A*_sum_ ̶ total area under curve. Within a cycle × site × cultivar combination, no overlap in letters indicates significant differences between storage temperatures, based on no overlap in the approximate 95% confidence intervals (data not shown). No statistical analysis was conducted for the parameters of *A*_1_, *A*_2_, *A*_3_, and *A*_sum_ due to a lack of replications after curve fitting. Right: yield formation analysis. CIR-cumulative intercepted radiation based on PAR, RUE-radiation use efficiency, Total DW-total dry weight, HI-harvest index, Tuber DW-tuber dry weight, Tuber DMC-tuber dry matter concentration in fraction, Yield-tuber total fresh weight. Within a cycle × site (S) × cultivar (CV) combination, no overlap in letters indicates significant differences between storage temperatures (ST) in post-hoc tests. No statistical analysis was conducted for the parameters CIR due to a lack of replications.

| **CV** | **ST** |  | **Canopy cover** | | | | | | | | |  | **Yield formation analysis** | | | | | | |
| --- | --- | --- | --- | --- | --- | --- | --- | --- | --- | --- | --- | --- | --- | --- | --- | --- | --- | --- | --- |
|  |  |  | *t*_m1_ | *t*_1_ | *t*_2_ | *t*_e_ | *v*_max_ | *A*_1_ | *A*_2_ | *A*_3_ | *A*_sum_ |  | CIR | RUE | Total DW | HI | Tuber DW | Tuber DMC | Yield |
|  | ˚C |  | d | d | d | d | % | % d | % d | % d | % d |  | MJ m^-2^ | g MJ^-1^ | g m^-2^ | g g^-1^ | g m^-2^ | g g^-1^ | kg m^-2^ |
| *Cycle 1 Est* | |  |  |  |  |  |  |  |  |  |  |  |  |  |  |  |  |  |  |
| **Agria** | 4 |  | 51.2 (0.6) a | 58.4 (1.4) a | 113.8 (2.5) a | 147.5 (0.8) a | 94.3 (1.6) a | 1100 | 5221 | 2173 | 8495 |  | 761.5 | 2.29 a | 1747 a | 0.75 a | 1311 a | 0.20 a | 6.5 a |
|  | 7 |  | 50.2 (0.8) a | 59.1 (1.7) a | 104.5 (3.0) a | 146.0 (0.9) a | 94.6 (1.9) a | 1299 | 4294 | 2662 | 8255 |  | 749.6 | 2.41 a | 1806 a | 0.75 a | 1341 a | 0.21 a | 6.5 a |
|  | 10 |  | 48.7 (0.7) a | 57.5 (1.5) a | 109.0 (3.0) a | 144.0 (1.3) a | 93.9 (1.6) a | 1271 | 4835 | 2244 | 8350 |  | 758.2 | 2.30 a | 1741 a | 0.64 a | 1113 a | 0.20 a | 5.6 a |
| **Festien** | 4 |  | 48.4 (0.4) a | 57.2 (1.0) a | 95.3 (11.0) a | 236.5^*^ (33.1) a | 95.5 (1.2) a | 1282 | 3637 | 3368 | 8286 |  | 855.0 | 2.22 a | 1897 a | 0.67 a | 1276 a | 0.29 a | 4.4 a |
|  | 7 |  | 49.8 (0.4) ab | 59.0 (0.8) a | 126.3 (4.4) b | 168.1 (7.2) a | 93.4 (0.8) a | 1303 | 6285 | 1407 | 8994 |  | 834.6 | 2.22 a | 1854 a | 0.66 a | 1207 a | 0.30 ab | 4.0 a |
|  | 10 |  | 50.1 (0.3) b | 59.3 (0.6) a | 132.4 (3.6) b | 165.2^*^  (6.8) a | 95.8 (0.6) a | 1337 | 7011 | 1047 | 9395 |  | 859.7 | 2.17 a | 1865 a | 0.71 a | 1309 a | 0.30 b | 4.3 a |
| **Innovator** | 4 |  | 49.2 (0.4) a | 57.7 (0.8) a | 92.5 (1.7) a | 129.0 (0.6) a | 98.6 (1.1) a | 1296 | 3433 | 2449 | 7178 |  | 685.8 | 2.60 a | 1782 a | 0.63 a | 1110 a | 0.20 ab | 5.5 a |
|  | 7 |  | 48.9 (0.6) a | 57.1 (1.4) a | 87.1 (3.4) a | 131.6 (1.4) a | 98.3 (2.1) a | 1256 | 2945 | 2955 | 7156 |  | 681.3 | 2.51 a | 1710 a | 0.63 a | 1048 a | 0.19 a | 5.5 a |
|  | 10 |  | 49.0 (0.7) a | 58.2 (1.5) a | 87.4 (3.4) a | 131.8 (1.4) a | 98.0 (2.0) a | 1370 | 2865 | 2939 | 7174 |  | 682.5 | 2.74 a | 1873 a | 0.60 a | 1115 a | 0.20 b | 5.5 a |
| **Lady Claire** | 4 |  | 49.7 (0.3) b | 57.0 (0.6) a | 95.2 (1.3) b | 123.8 (0.5) a | 98.7 (0.9) a | 1147 | 3777 | 1938 | 6861 |  | 664.1 | 2.41 a | 1602 a | 0.63 a | 1003 a | 0.22 a | 4.5 a |
|  | 7 |  | 49.1 (0.5) b | 56.7 (1.0) a | 83.9 (2.1) a | 121.6 (0.7) a | 99.3 (1.7) a | 1193 | 2704 | 2544 | 6441 |  | 628.5 | 2.33 a | 1467 a | 0.61 a | 902 a | 0.23 a | 4.0 a |
|  | 10 |  | 47.1 (0.4) a | 54.6 (1.0) a | 80.5 (1.9) a | 122.6 (0.7) a | 99.3 (1.4) a | 1179 | 2566 | 2825 | 6570 |  | 639.6 | 2.33 a | 1491 a | 0.68 a | 1017 a | 0.22 a | 4.5 a |
| *Cycle 1 Wanroij* | |  |  |  |  |  |  |  |  |  |  |  |  |  |  |  |  |  |  |
| **Agria** | 4 |  | 41.4 (0.2) a | 47.1 (0.5) a | 101.2 (1.0) a | 132.4 (0.4) a | 94.5 (0.7) a | 875 | 5108 | 2005 | 7988 |  | 723.5 | 2.58 a | 1867 a | 0.78 a | 1447 a | 0.21 a | 7.0 a |
|  | 7 |  | 40.5 (0.3) a | 47.1 (0.6) a | 102.5 (1.0) a | 132.4 (0.4) a | 95.7 (0.7) a | 989 | 5301 | 1950 | 8240 |  | 745.2 | 3.03 a | 2259 ab | 0.69 a | 1534 a | 0.21 a | 7.4 ab |
|  | 10 |  | 41.3 (0.3) a | 47.8 (0.7) a | 100.9 (1.3) a | 137.5 (0.4) b | 95.2 (0.9) a | 977 | 5054 | 2354 | 8385 |  | 753.1 | 2.92 a | 2197 b | 0.77 a | 1676 a | 0.20 a | 8.3 b |
| **Festien** | 4 |  | 41.7 (0.2) a | 48.9 (0.5) a | 100.8 (3.4) a | 178.4 (6.8) a | 96.7 (0.6) a | 1079 | 5016 | 3048 | 9143 |  | 851.8 | 2.56 a | 2177 a | 0.71 a | 1549 a | 0.30 a | 5.2 a |
|  | 7 |  | 41.6 (0.3) a | 48.4 (0.7) a | 107.7 (3.9) a | 164.7^*^ (6.2) a | 95.6 (0.9) a | 1011 | 5670 | 2593 | 9273 |  | 841.0 | 2.65 a | 2227 a | 0.62 a | 1319 a | 0.29 a | 4.5 a |
|  | 10 |  | 41.8 (0.4) a | 49.6 (0.8) a | 106.2 (4.2) a | 166.6^*^ (6.8) a | 96.7 (0.9) a | 1144 | 5471 | 2717 | 9333 |  | 849.9 | 2.49 a | 2116 a | 0.67 a | 1415 a | 0.30 a | 4.7 a |
| **Innovator** | 4 |  | 40.8 (0.4) a | 48.3 (0.9) a | 74.9 (1.9) b | 119.2 (0.7) a | 96.6 (1.6) a | 1104 | 2576 | 2864 | 6544 |  | 627.3 | 2.69 a | 1685 a | 0.71 a | 1190 a | 0.20 a | 6.1 a |
|  | 7 |  | 40.5 (0.3) a | 48.5 (0.7) a | 66.8 (1.7) a | 118.5 (0.5) a | 98.0 (1.4) a | 1185 | 1795 | 3365 | 6345 |  | 609.8 | 2.51 a | 1529 a | 0.75 a | 1133 a | 0.20 a | 5.8 a |
|  | 10 |  | 40.5 (0.8) a | 48.2 (1.6) a | 61.9 (4.4) a | 115.9 (1.7) a | 97.9 (3.5) a | 1140 | 1341 | 3503 | 5984 |  | 578.4 | 3.10 a | 1794 a | 0.60 a | 1065 a | 0.19 a | 5.5 a |
| **Lady Claire** | 4 |  | 40.4 (0.5) a | 46.8 (1.0) a | 63.8 (2.4) a | 104.8 (0.8) a | 99.0 (2.2) a | 999 | 1683 | 2724 | 5405 |  | 528.5 | 3.01 a | 1593 a | 0.67 a | 1061 a | 0.22 a | 4.9 a |
|  | 7 |  | 41.0 (0.6) a | 46.8 (1.3) a | 62.1 (3.4) a | 104.3 (1.1) a | 99.5 (3.2) a | 921 | 1528 | 2812 | 5261 |  | 514.6 | 2.72 a | 1401 a | 0.72 a | 997 a | 0.21 a | 4.7 a |
|  | 10 |  | 40.2 (0.9) a | 46.4 (1.9) a | 66.8 (4.6) a | 101.0 (1.8) a | 97.7 (4.0) a | 961 | 1991 | 2262 | 5214 |  | 509.4 | 2.70 a | 1374 a | 0.75 a | 1021 a | 0.21 a | 4.8 a |
| *Cycle 2 Est* | |  |  |  |  |  |  |  |  |  |  |  |  |  |  |  |  |  |  |
| **Agria** | 4 |  | 54.8 (0.6) a | 63.9 (1.1) a | 116.6 (1.1) b | 141.5 (0.4) a | 98.0 (0.9) a | 1392 | 5163 | 1692 | 8246 |  | 641.1 | 2.45 a | 1572 a | 0.62 a | 973 a | 0.23 a | 4.2 a |
|  | 7 |  | 55.2 (0.7) a | 64.1 (1.2) a | 106.3 (1.6) a | 142.2 (0.5) a | 97.3 (1.1) a | 1356 | 4106 | 2378 | 7839 |  | 613.0 | 2.52 a | 1544 a | 0.71 a | 1089 a | 0.23 a | 4.7 ab |
|  | 10 |  | 55.0 (0.6) a | 64.1 (1.1) a | 108.9 (1.4) a | 142.1 (0.5) a | 98.2 (1.0) a | 1399 | 4398 | 2233 | 8031 |  | 627.1 | 2.29 a | 1436 a | 0.80 a | 1149 a | 0.23 a | 5.0 b |
| **Festien** | 4 |  | 55.3 (0.7) a | 66.4 (1.2) a | 103.5 (11.1) a | 213.5^*^  (36.2) a | 98.6 (1.2) a | 1636 | 3663 | 2596 | 7896 |  | 688.7 | 2.25 a | 1548 a | 0.80 a | 1238 a | 0.33 a | 3.7 a |
|  | 7 |  | 55.8 (0.4) a | 66.1 (0.7) a | 105.6 (13.7) a | 264.5^*^  (82.6) a | 98.7 (0.8) a | 1552 | 3900 | 2462 | 7914 |  | 689.3 | 2.20 a | 1516 a | 0.75 a | 1123 a | 0.33 a | 3.4 a |
|  | 10 |  | 56.8 (0.5) a | 66.8 (0.8) a | 107.2 (12.2) a | 235.4^*^  (58.0) a | 98.6 (0.9) a | 1516 | 3984 | 2356 | 7857 |  | 677.9 | 2.34 a | 1585 a | 0.73 a | 1136 a | 0.33 a | 3.4 a |
| **Innovator** | 4 |  | 50.9 (2.5) a | 57.8 (5.4) a | 81.7 (3.5) ab | 126.4 (1.1) b | 97.6 (2.7) a | 1081 | 2333 | 2945 | 6359 |  | 517.5 | 2.29 a | 1188 a | 0.84 a | 989 a | 0.22 a | 4.5 a |
|  | 7 |  | 52.1 (2.7) a | 59.1 (5.5) a | 93.9 (2.7) b | 121.5 (1.0) a | 96.5 (1.9) a | 1094 | 3355 | 1833 | 6283 |  | 511.1 | 2.37 a | 1211 a | 0.80 a | 976 a | 0.22 a | 4.4 a |
|  | 10 |  | 52.0 (2.6) a | 59.2 (5.4) a | 81.2 (2.9) a | 126.0 (0.8) b | 97.9 (2.2) a | 1138 | 2154 | 2961 | 6254 |  | 508.2 | 2.39 a | 1213 a | 0.74 a | 892 a | 0.22 a | 4.0 a |
| **Lady Claire** | 4 |  | 51.4 (5.2) a | 56.8 (10) a | 68.3 (6.8) a | 118.4 (1.7) a | 98.6 (5.4) a | 899 | 1131 | 3318 | 5348 |  | 438.4 | 2.15 a | 941 a | 0.60 a | 550 a | 0.22 a | 2.5 a |
|  | 7 |  | 50.7 (2.0) a | 56.8 (4.1) a | 82.0 (3.0) a | 116.7 (1.5) a | 97.4 (2.1) a | 978 | 2445 | 2307 | 5730 |  | 472.5 | 2.28 a | 1076 a | 0.77 a | 770 a | 0.22 a | 3.5 a |
|  | 10 |  | 51.0 (4.0) a | 56.7 (8.0) a | 68.8 (6.3) a | 119.0 (1.7) a | 99.4 (5.0) a | 945 | 1195 | 3348 | 5488 |  | 450.9 | 2.22 a | 1003 a | 0.70 a | 701 a | 0.21 a | 3.3 a |
| *Cycle 2 Nagele* | |  |  |  |  |  |  |  |  |  |  |  |  |  |  |  |  |  |  |
| **Agria** | 4 |  | 61.7 (0.9) a | 73.7 (1.8) a | 121.3 (2.2) a | 146.9 (1.0) a | 97.4 (1.4) a | 1766 | 4632 | 1476 | 7874 |  | 635.2 | 2.71 a | 1720 a | 0.68 a | 1231 a | 0.21 a | 5.7 a |
|  | 7 |  | 61.7 (0.6) a | 71.8 (1.2) a | 124.4 (1.6) a | 145.9 (0.6) a | 97.7 (1.1) a | 1550 | 5135 | 1273 | 7959 |  | 635.5 | 2.25 a | 1431 a | 0.80 a | 1126 a | 0.21 a | 5.4 a |
|  | 10 |  | 63.6 (0.4) a | 75.3 (1.0) a | 127.2 (1.3) a | 147.6 (0.7) a | 98.1 (0.7) a | 1757 | 5089 | 1091 | 7937 |  | 634.8 | 2.64 a | 1674 a | 0.74 a | 1225 a | 0.21 a | 5.7 a |
| **Festien** | 4 |  | 61.8 (0.4) a | 77.4 (0.9) a | 103.3 (7.3) a | 231.9^*^  (25.8) a | 98.6 (0.7) a | 2190 | 2554 | 2688 | 7433 |  | 666.3 | 2.45 a | 1632 a | 0.70 a | 1137 a | 0.30 a | 3.8 a |
|  | 7 |  | 64.5 (0.4) b | 78.4 (0.9) a | 120.2 (24.7) a | 291.6^*^ (270.0) a | 98.8 (0.7) a | 2021 | 4133 | 1574 | 7728 |  | 649.9 | 2.30 a | 1492 a | 0.74 a | 1031 a | 0.29 a | 3.5 a |
|  | 10 |  | 65.1 (0.3) b | 79.3 (0.6) a | 100.8 (14.8) a | 340.1^*^ (111.2) a | 99.1 (0.7) a | 2071 | 2134 | 2870 | 7075 |  | 644.7 | 2.28 a | 1472 a | 0.79 a | 1148 a | 0.30 a | 3.8 a |
| **Innovator** | 4 |  | 59.2 (0.7) a | 72.1 (1.4) a | 112.6 (1.4) b | 135.5 (0.4) a | 97.3 (1.2) a | 1849 | 3940 | 1553 | 7342 |  | 599.2 | 2.09 a | 1255 a | 0.90 a | 1134 a | 0.22 a | 5.2 a |
|  | 7 |  | 60.3 (1.2) a | 74.5 (2.7) a | 103.1 (2.7) a | 134.9 (0.7) a | 97.0 (2.3) a | 1988 | 2778 | 2132 | 6899 |  | 568.3 | 2.29 ab | 1301 a | 0.83 a | 1076 a | 0.22 a | 4.9 a |
|  | 10 |  | 62.8 (1.7) a | 78.8 (3.8) a | 93.7 (4.5) a | 134.6 (1.0) a | 98.4 (4.0) a | 2241 | 1461 | 2764 | 6467 |  | 536.6 | 2.64 b | 1414 a | 0.73 a | 1030 a | 0.22 a | 4.6 a |
| **Lady Claire** | 4 |  | 58.3 (0.4) a | 67.8 (0.7) a | 104.3 (0.9) b | 122.5 (0.3) a | 96.8 (0.8) a | 1439 | 3528 | 1233 | 6200 |  | 521.5 | 2.54 a | 1324 a | 0.69 a | 916 a | 0.21 a | 4.3 a |
|  | 7 |  | 58.1 (0.6) a | 68.1 (1.0) a | 105.1 (1.1) b | 122.3 (0.3) a | 96.0 (1.1) a | 1476 | 3556 | 1157 | 6189 |  | 521.0 | 2.12 a | 1105 a | 0.89 a | 970 a | 0.22 a | 4.4 a |
|  | 10 |  | 58.9 (1.0) a | 69.5 (1.9) a | 92.5 (2.8) a | 128.2 (0.7) b | 99.4 (2.7) a | 1619 | 2290 | 2436 | 6344 |  | 531.8 | 2.41 a | 1284 a | 0.71 a | 898 a | 0.22 a | 4.1 a |
| *Cycle 2 Wanroij* | |  |  |  |  |  |  |  |  |  |  |  |  |  |  |  |  |  |  |
| **Agria** | 4 |  | 43.4 (0.2) a | 49.6 (0.5) a | 108.8 (0.9) a | 135.3 (0.3) a | 99.0 (0.8) a | 983 | 5861 | 1799 | 8643 |  | 688.7 | 2.64 a | 1815 a | 0.88 a | 1568 a | 0.21 a | 7.4 a |
|  | 7 |  | 44.7 (0.3) b | 51.5 (0.8) a | 110.1 (1.3) a | 136.0 (0.5) a | 98.6 (1.0) a | 1052 | 5782 | 1756 | 8591 |  | 681.0 | 2.72 a | 1852 a | 0.81 a | 1498 a | 0.21 a | 7.1 a |
|  | 10 |  | 44.1 (0.3) ab | 50.9 (0.7) a | 108.5 (1.3) a | 135.2 (0.4) a | 98.6 (1.1) a | 1051 | 5682 | 1806 | 8539 |  | 679.9 | 2.90 a | 1969 a | 0.75 a | 1437 a | 0.20 a | 7.0 a |
| **Festien** | 4 |  | 42.9 (0.2) a | 50.6 (0.4) a | 109.4 (9.6) a | 284.6^*^ (59.4) a | 99.3 (0.6) a | 1173 | 5835 | 2782 | 9790 |  | 823.5 | 2.79 a | 2294 a | 0.78 a | 1787 a | 0.31 a | 5.8 a |
|  | 7 |  | 44.8 (0.2) b | 51.9 (0.5) ab | 109.1 (7.7) a | 255.8^*^ (36.8) a | 99.1 (0.6) a | 1109 | 5667 | 2799 | 9574 |  | 799.2 | 2.84 a | 2267 a | 0.80 a | 1811 a | 0.31 a | 5.9 a |
|  | 10 |  | 44.8 (0.1) b | 52.2 (0.3) b | 120.9 (6.8) a | 271.5^*^ (54.7) a | 99.4 (0.3) a | 1141 | 6830 | 2046 | 10017 |  | 807.9 | 3.03 a | 2452 a | 0.72 a | 1768 a | 0.31 a | 5.7 a |
| **Innovator** | 4 |  | 42.1 (0.6) a | 50.2 (1.3) a | 93.9 (2.7) a | 123.7 (1.1) a | 97.4 (2.1) a | 1191 | 4262 | 1980 | 7432 |  | 613.6 | 3.26 a | 1999 a | 0.70 a | 1392 a | 0.22 a | 6.3 a |
|  | 7 |  | 43.4 (0.4) a | 51.3 (0.9) a | 92.2 (1.8) a | 127.1 (0.6) ab | 97.7 (1.5) a | 1179 | 3992 | 2317 | 7488 |  | 613.4 | 2.78 a | 1708 a | 0.73 a | 1250 a | 0.22 a | 5.7 a |
|  | 10 |  | 42.9 (0.3) a | 50.7 (0.6) a | 92.9 (1.3) a | 129.1 (0.5) b | 98.6 (0.9) a | 1185 | 4161 | 2417 | 7763 |  | 635.1 | 2.90 a | 1843 a | 0.74 a | 1340 a | 0.22 a | 6.1 a |
| **Lady Claire** | 4 |  | 42.3 (0.4) a | 48.5 (0.8) a | 89.4 (1.7) a | 113.6 (0.5) a | 96.4 (1.6) a | 943 | 3947 | 1604 | 6495 |  | 545.0 | 2.63 a | 1434 a | 0.84 a | 1187 a | 0.23 a | 5.2 a |
|  | 7 |  | 42.8 (0.3) a | 49.3 (0.6) a | 86.1 (1.3) a | 112.7 (0.4) a | 98.0 (1.3) a | 1009 | 3611 | 1787 | 6407 |  | 539.1 | 2.70 a | 1456 a | 0.76 a | 1101 a | 0.23 a | 4.8 a |
|  | 10 |  | 42.5 (0.5) a | 49.1 (0.9) a | 85.8 (1.8) a | 112.7 (0.5) a | 97.1 (1.8) a | 1016 | 3564 | 1790 | 6370 |  | 537.2 | 2.78 a | 1491 a | 0.73 a | 1091 a | 0.22 a | 4.9 a |
| *Cycle 3 Est* | |  |  |  |  |  |  |  |  |  |  |  |  |  |  |  |  |  |  |
| **Agria** | 4 |  | 53.5 (0.4) a | 64.2 (1.0) a | 105.3 (1.7) a | 144.9 (0.6) b | 94.6 (1.0) a | 1516 | 3888 | 2556 | 7960 |  | 761.2 | 2.65 a | 2015 a | 0.68 a | 1359 a | 0.23 a | 6.0 a |
|  | 10 |  | 53.4 (0.8) a | 65.4 (2.0) a | 104.1 (2.9) a | 142.2 (0.7) ab | 94.5 (1.9) a | 1661 | 3654 | 2460 | 7775 |  | 752.0 | 2.44 a | 1834 a | 0.68 a | 1244 a | 0.24 a | 5.3 a |
|  | 17 |  | 53.7 (0.5) a | 64.0 (1.3) a | 103.9 (2.0) a | 139.7 (0.6) a | 96.4 (1.3) a | 1500 | 3850 | 2363 | 7712 |  | 751.0 | 2.43 a | 1827 a | 0.62 a | 1145 a | 0.23 a | 5.0 a |
| **Festien** | 4 |  | 54.1 (0.3) a | 66.8 (0.8) a | 132.7 (2.9) b | 153.4^*^ (5.4) a | 94.7 (0.6) a | 1738 | 6239 | 1017 | 8994 |  | 828.6 | 2.37 a | 1961 a | 0.68 a | 1327 a | 0.31 a | 4.3 a |
|  | 10 |  | 54.3 (0.4) a | 68.4 (1.1) a | 113.4 (14.1) ab | 223.0^*^ (62.7) a | 95.3 (0.9) a | 1905 | 4288 | 2266 | 8460 |  | 839.8 | 2.31 a | 1937 a | 0.70 a | 1353 a | 0.30 a | 4.5 a |
|  | 17 |  | 53.0 (0.4) a | 66.5 (1.1) a | 109.2 (7.9) a | 195.4^*^ (20.1) a | 95.6 (1.0) a | 1832 | 4086 | 2536 | 8454 |  | 846.4 | 2.33 a | 1973 a | 0.70 a | 1373 a | 0.31 a | 4.4 a |
| **Innovator** | 4 |  | 55.8 (0.3) b | 68.8 (0.8) a | 88.5 (1.0) a | 117.1 (0.2) ab | 93.4 (0.9) a | 1761 | 1839 | 1847 | 5447 |  | 567.5 | 2.36 a | 1338 a | 0.83 a | 1108 a | 0.24 a | 4.6 a |
|  | 10 |  | 53.8 (0.4) a | 67.1 (1.0) a | 85.7 (1.4) a | 116.7 (0.3) a | 92.9 (1.2) a | 1775 | 1723 | 1988 | 5486 |  | 574.3 | 2.57 a | 1478 a | 0.74 a | 1086 a | 0.23 a | 4.7 a |
|  | 17 |  | 55.7 (0.4) b | 67.0 (0.9) a | 86.9 (1.5) a | 118.2 (0.4) b | 89.7 (1.2) a | 1521 | 1779 | 1935 | 5236 |  | 544.5 | 2.42 a | 1318 a | 0.98 a | 1274 a | 0.23 a | 5.5 a |
| **Lady Claire** | 4 |  | 52.7 (0.4) b | 62.9 (1.0) a | 80.0 (2.6) b | 112.7 (1.3) c | 93.2 (1.3) a | 1438 | 1595 | 2093 | 5126 |  | 542.9 | 2.56 a | 1391 b | 0.79 a | 1103 a | 0.25 a | 4.4 a |
|  | 10 |  | 50.5 (0.4) a | 61.3 (0.8) a | 82.8 (1.5) b | 108.8 (0.5) b | 96.4 (1.1) a | 1543 | 2064 | 1735 | 5342 |  | 570.3 | 2.33 a | 1329 ab | 0.68 a | 913 a | 0.25 a | 3.7 a |
|  | 17 |  | 50.4 (0.4) a | 62.5 (0.9) a | 69.8 (1.6) a | 106.3 (0.4) a | 95.2 (1.3) a | 1662 | 694 | 2372 | 4729 |  | 509.1 | 2.26 a | 1149 a | 0.73 a | 840 a | 0.25 a | 3.4 a |
| *Cycle 3 Nagele* | |  |  |  |  |  |  |  |  |  |  |  |  |  |  |  |  |  |  |
| **Agria** | 4 |  | 49.2 (0.7) a | 61.4 (1.8) a | 95.7 (2.5) a | 132.2 (0.8) a | 97.8 (1.7) a | 1705 | 3360 | 2439 | 7503 |  | 736.6 | 2.59 a | 1909 a | 0.73 a | 1390 ab | 0.24 a | 5.8 a |
|  | 10 |  | 49.0 (0.9) a | 61.1 (2.1) a | 97.6 (3.0) a | 132.9 (1.0) a | 96.4 (1.9) a | 1669 | 3523 | 2328 | 7520 |  | 736.0 | 2.77 a | 2037 a | 0.71 a | 1444 b | 0.25 a | 5.9 a |
|  | 17 |  | 54.3 (0.4) b | 67.8 (1.0) b | 98.7 (1.5) a | 133.9 (0.5) a | 97.6 (1.0) a | 1880 | 3020 | 2357 | 7256 |  | 702.0 | 2.87 a | 2016 a | 0.63 a | 1264 a | 0.24 a | 5.2 a |
| **Festien** | 4 |  | 48.3 (0.4) a | 63.3 (1.0) a | 109.2 (3.9) a | 169.0^*^ (6.8) a | 97.7 (0.8) a | 1981 | 4486 | 2005 | 8473 |  | 850.8 | 2.48 a | 2108 a | 0.68 a | 1441 a | 0.32 a | 4.5 b |
|  | 10 |  | 48.8 (0.5) a | 61.1 (1.2) a | 108.8 (3.9) a | 159.3 (4.9) a | 97.8 (1.0) a | 1717 | 4669 | 2032 | 8418 |  | 840.2 | 2.38 a | 2000 a | 0.70 a | 1403 a | 0.32 a | 4.4 ab |
|  | 17 |  | 48.7 (0.4) a | 62.7 (0.9) a | 101.5 (4.5) a | 179.5^*^ (8.4) a | 98.4 (0.8) a | 1904 | 3813 | 2519 | 8237 |  | 848.0 | 2.43 a | 2058 a | 0.64 a | 1324 a | 0.32 a | 4.1 a |
| **Innovator** | 4 |  | 54.1 (0.3) a | 68.3 (0.8) a | 87.6 (1.0) b | 117.2 (0.3) a | 98.1 (0.8) a | 1967 | 1894 | 2004 | 5865 |  | 590.8 | 2.45 a | 1450 a | 0.85 a | 1239 a | 0.24 a | 5.2 a |
|  | 10 |  | 53.5 (0.5) a | 69.5 (1.2) ab | 84.4 (1.3) ab | 116.4 (0.3) a | 97.4 (1.2) a | 2132 | 1452 | 2153 | 5737 |  | 580.2 | 2.60 a | 1509 a | 0.78 a | 1159 a | 0.23 a | 5.0 a |
|  | 17 |  | 57.7 (0.7) b | 74.3 (1.7) b | 80.4 (2.2) a | 122.2 (0.5) b | 98.5 (1.9) a | 2262 | 595 | 2819 | 5677 |  | 567.7 | 2.77 a | 1571 a | 0.73 a | 1141 a | 0.24 a | 4.8 a |
| **Lady Claire** | 4 |  | 49.0 (0.3) b | 60.5 (0.8) a | 84.2 (1.0) a | 115.2 (0.2) b | 97.9 (0.9) a | 1632 | 2327 | 2081 | 6040 |  | 615.4 | 2.49 a | 1531 b | 0.80 a | 1232 b | 0.26 b | 4.7 b |
|  | 10 |  | 46.4 (0.4) a | 58.1 (1.0) a | 89.0 (1.3) b | 108.0 (0.5) a | 98.7 (1.0) a | 1656 | 3048 | 1306 | 6010 |  | 620.2 | 2.51 a | 1555 b | 0.77 a | 1199 b | 0.25 ab | 4.7 b |
|  | 17 |  | 44.9 (0.8) a | 58.0 (1.8) a | 79.3 (2.4) a | 106.3 (0.6) a | 97.2 (1.8) a | 1758 | 2073 | 1807 | 5638 |  | 584.4 | 2.15 a | 1256 a | 0.84 a | 1039 a | 0.25 a | 4.2 a |
| *Cycle 3 Wanroij* | |  |  |  |  |  |  |  |  |  |  |  |  |  |  |  |  |  |  |
| **Agria** | 4 |  | 43.2 (0.7) a | 54.8 (1.5) a | 81.8 (2.4) a | 114.1 (0.7) a | 91.9 (1.8) a | 1497 | 2480 | 2026 | 6002 |  | 629.3 | 2.63 a | 1654 a | 0.71 a | 1172 a | 0.22 a | 5.2 a |
|  | 10 |  | 41.9 (0.4) a | 53.3 (0.9) a | 87.1 (1.3) a | 114.7 (0.4) a | 95.8 (0.9) a | 1529 | 3237 | 1815 | 6581 |  | 687.6 | 2.69 ab | 1852 ab | 0.69 a | 1266 a | 0.23 a | 5.6 a |
|  | 17 |  | 45.6 (0.4) b | 55.0 (0.7) a | 83.9 (1.5) a | 114.7 (0.5) a | 96.3 (1.1) a | 1356 | 2778 | 2032 | 6166 |  | 643.8 | 3.09 b | 1990 b | 0.65 a | 1288 a | 0.23 a | 5.6 a |
| **Festien** | 4 |  | 40.9 (0.6) a | 53.5 (1.5) a | 81.3 (2.4) a | 128.7 (0.8) b | 97.6 (1.5) a | 1666 | 2713 | 3105 | 7484 |  | 766.1 | 2.74 a | 2101 a | 0.61 a | 1283 a | 0.32 ab | 4.0 a |
|  | 10 |  | 42.9 (0.5) a | 55.5 (1.2) a | 83.5 (2.2) a | 128.2 (0.7) b | 97.2 (1.4) a | 1691 | 2723 | 2928 | 7342 |  | 750.0 | 2.69 a | 2016 a | 0.67 a | 1349 a | 0.34 b | 4.0 a |
|  | 17 |  | 41.3 (0.5) a | 52.5 (1.3) a | 89.0 (2.2) a | 123.6 (0.8) a | 97.4 (1.2) a | 1528 | 3558 | 2293 | 7380 |  | 759.8 | 2.59 a | 1967 a | 0.63 a | 1244 a | 0.32 a | 3.9 a |
| **Innovator** | 4 |  | 48.3 (0.8) a | 64.7 (1.8) a | 87.8 (2.2) a | 103.1 (0.5) a | 83.9 (1.8) b | 1828 | 1941 | 899 | 4669 |  | 497.2 | 2.59 b | 1288 b | 0.72 a | 925 b | 0.22 a | 4.1 b |
|  | 10 |  | 46.8 (0.7) a | 61.0 (1.5) a | 85.7 (2.1) a | 104.1 (0.7) a | 91.8 (1.7) c | 1779 | 2266 | 1178 | 5223 |  | 555.9 | 2.04 a | 1134 b | 0.83 a | 944 b | 0.22 a | 4.3 b |
|  | 17 |  | 46.8 (1.9) a | 63.6 (4.5) a | 85.0 (5.6) a | 104.3 (1.8) a | 54.6 (2.8) a | 1203 | 1167 | 735 | 3104 |  | 330.2 | 2.46 b | 812 a | 0.84 a | 683 a | 0.23 a | 3.0 a |
| **Lady Claire** | 4 |  | 42.5 (0.4) b | 51.2 (0.9) a | 78.7 (1.1) c | 101.4 (0.2) a | 96.5 (0.9) a | 1252 | 2657 | 1511 | 5420 |  | 582.4 | 2.49 a | 1451 a | 0.78 a | 1123 a | 0.24 a | 4.6 a |
|  | 10 |  | 40.8 (0.4) a | 50.3 (1.1) a | 73.2 (1.2) b | 101.8 (0.3) a | 97.8 (0.9) a | 1345 | 2248 | 1911 | 5505 |  | 592.0 | 2.44 a | 1442 a | 0.78 a | 1122 a | 0.23 a | 4.8 a |
|  | 17 |  | 41.4 (0.6) ab | 52.5 (1.5) a | 65.3 (2.5) a | 99.1 (1.2) a | 96.1 (1.8) a | 1502 | 1232 | 2211 | 4944 |  | 533.6 | 2.62 a | 1398 a | 0.73 a | 1021 a | 0.23 a | 4.3 a |

^*^These crops did not senesce (fully) within the time frame of the experiment. The average canopy cover across three blocks at the end of *P*3 was above 75%

**Table S5** ANOVA results of yield formation analysis per variable. The analysis includes CIR-cumulative intercepted radiation based on PAR, RUE-radiation use efficiency, Total DW-total dry weight production, HI-harvest index, Tuber DW-tuber dry weight, Tuber DMC-tuber dry matter concentration in fraction, and Yield. *p*-values < 0.05 are in bold. Within a cycle × site (S) × cultivar (CV), no overlap in letters indicates significant differences between storage temperatures (ST) in Tucky’s post-hoc tests. No statistical analysis of ST × CV × S interaction on CIR due to lack of replications. Details of yield formation analysis are listed in Table S4

| **Cycle** |  | **CIR** | **RUE** | **Total DW** | **HI** | **Tuber DW** | **Tuber DMC** | **Yield** |
| --- | --- | --- | --- | --- | --- | --- | --- | --- |
|  |  | MJ m^-2^ | g MJ^-1^ | g m^-2^ | g g^-1^ | g m^-2^ | g g^-1^ | kg m^-2^ |
| **Cycle 1** |  |  |  |  |  |  |  |  |
| ST |  | 0.201 | 0.813 | 0.995 | 0.798 | 0.303 | 0.904 | 0.410 |
| CV |  | **< 0.001** | **0.001** | **< 0.001** | 0.095 | **< 0.001** | **< 0.001** | **< 0.001** |
| S |  | **< 0.001** | **< 0.001** | 0.451 | 0.543 | 0.116 | 0.386 | 0.058 |
| ST × CV |  | 0.270 | 0.078 | **0.023** | 0.391 | 0.607 | 0.101 | 0.665 |
| ST × S |  | 0.388 | 0.744 | 0.651 | 0.972 | 0.902 | 0.114 | 0.624 |
| CV × S |  | **< 0.001** | 0.455 | **< 0.001** | 0.559 | **0.001** | **0.005** | **0.007** |
| ST × CV × S |  |  | 0.631 | 0.467 | 0.551 | 0.059 | 0.066 | **0.013** |
| **Cycle 2** |  |  |  |  |  |  |  |  |
| ST |  | 0.303 | 0.124 | 0.130 | 0.238 | 0.282 | 0.925 | 0.309 |
| CV |  | **< 0.001** | 0.197 | **< 0.001** | 0.846 | **< 0.001** | **< 0.001** | **< 0.001** |
| S |  | **< 0.001** | **0.024** | **< 0.001** | 0.935 | **< 0.001** | 0.485 | **< 0.001** |
| ST × CV |  | 0.732 | 1.000 | 0.975 | 0.757 | 0.704 | 0.486 | 0.313 |
| ST × S |  | 0.699 | 0.497 | 0.264 | 0.554 | 0.592 | 0.364 | 0.329 |
| CV × S |  | **< 0.001** | 0.300 | **< 0.001** | 0.232 | **< 0.001** | **< 0.001** | **< 0.001** |
| ST × CV × S |  |  | 0.128 | 0.423 | 0.267 | 0.370 | 0.991 | 0.153 |
| **Cycle 3** |  |  |  |  |  |  |  |  |
| ST |  | **0.025** | 0.302 | **0.016** | 0.174 | **< 0.001** | 0.329 | **< 0.001** |
| CV |  | **< 0.001** | **< 0.001** | **< 0.001** | **< 0.001** | **< 0.001** | **< 0.001** | **< 0.001** |
| S |  | **< 0.001** | 0.461 | 0.109 | 0.856 | 0.298 | 0.269 | 0.802 |
| ST × CV |  | 0.279 | 0.258 | **0.005** | 0.324 | 0.383 | 0.057 | 0.544 |
| ST × S |  | 0.347 | **0.041** | 0.789 | 0.202 | 0.379 | 0.950 | 0.258 |
| CV × S |  | **0.038** | **< 0.001** | **< 0.001** | 0.207 | **< 0.001** | **< 0.001** | **< 0.001** |
| ST × CV × S |  |  | **0.002** | **< 0.001** | 0.650 | 0.114 | 0.162 | 0.057 |

**
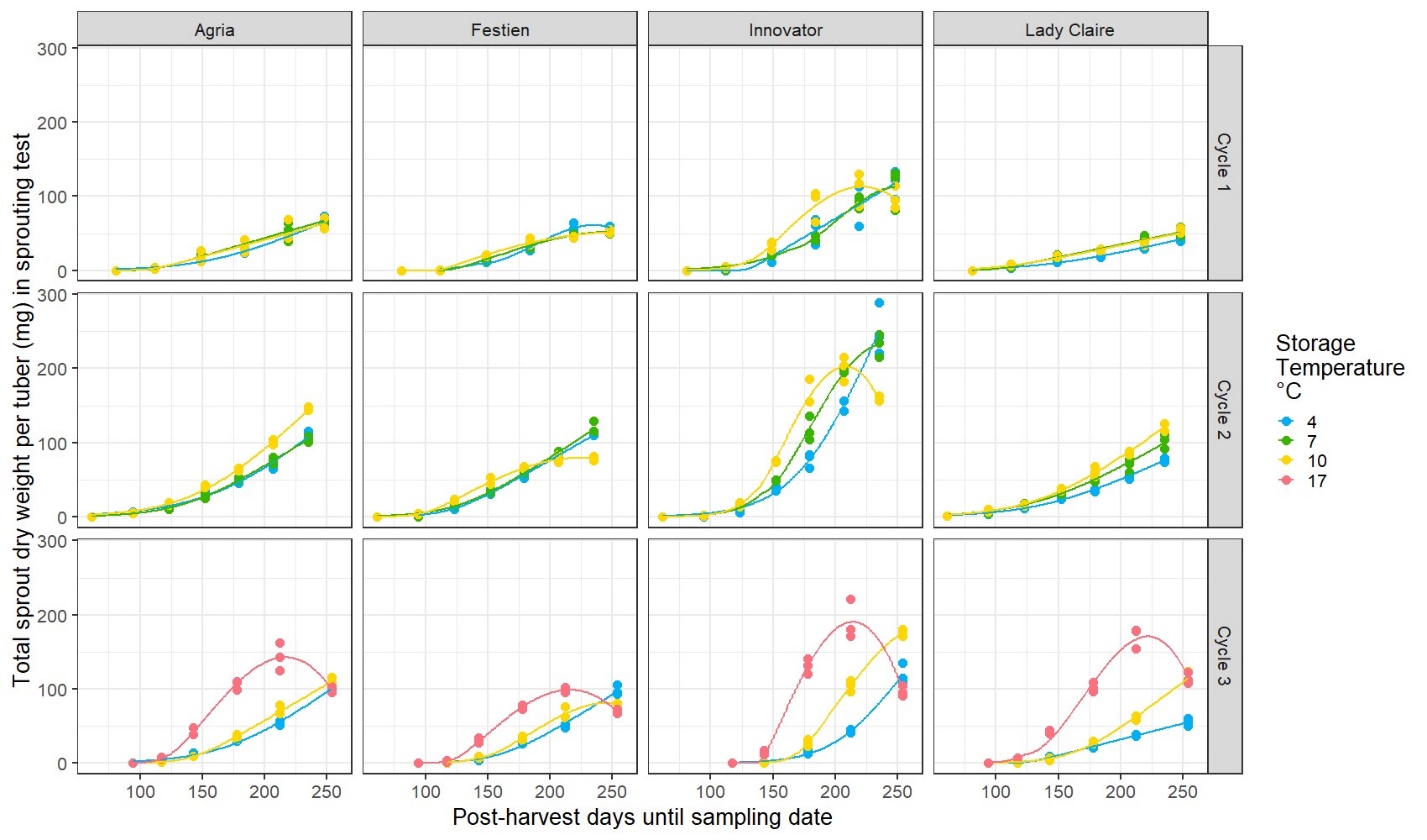
**

**Fig. S1** Observations and fitted curves of total sprout dry weight per tuber (mg) produced in sprouting tests carried out after different sampling dates during storage of seed tubers of four cultivars stored at different temperatures (in colours) during the storage seasons in three cycles. Points refer to the sprout dry weight per tuber produced in 2 (Cycle 1) or 3 (Cycles 2 and 3) weeks at 18 ˚C in darkness. Sprouts produced during storage were removed before the test. Figure from Zou et al. (2024)


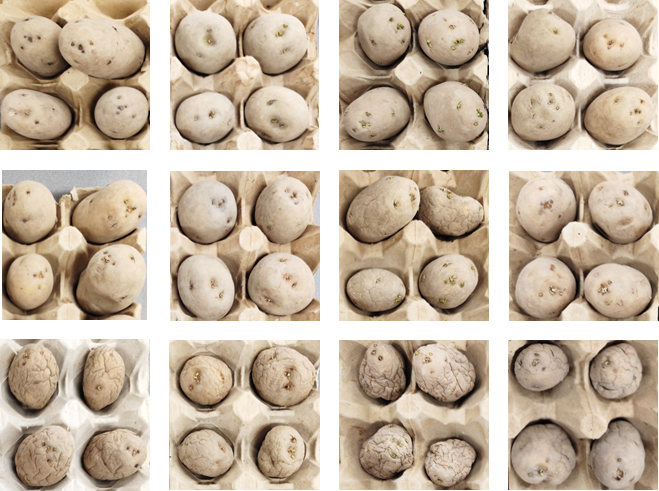


**Fig. S2** Example of the physical appearance of potato seed tubers at planting time of four cultivars Agria, Festien, Innovator, and Lady Claire (from left to right) stored at 4 ˚C, 10 ˚C, and 17 ˚C (from top to bottom). Pictures were taken in Cycle 3 (May 2022)

**
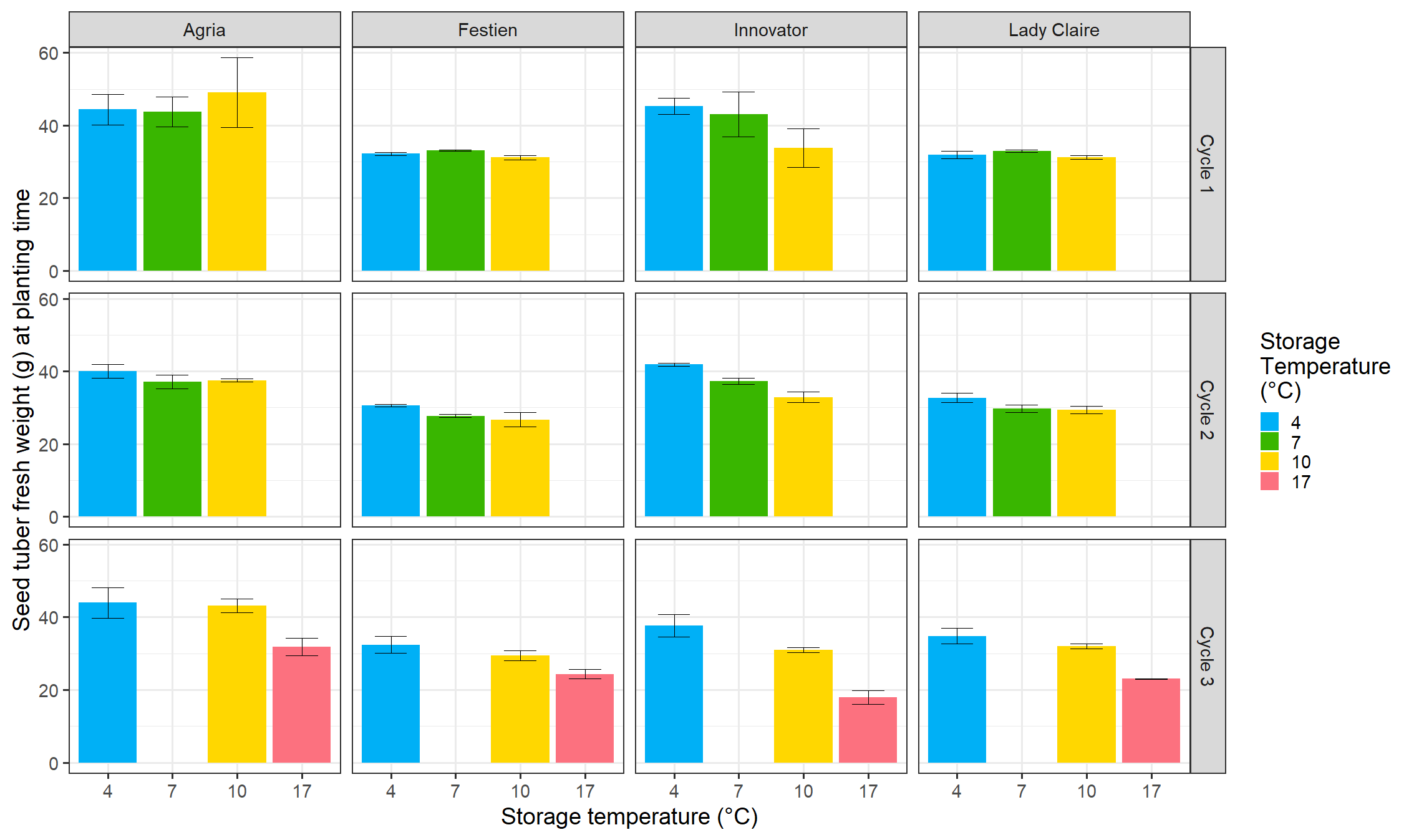
**

**Fig. S3** Average fresh weight (g) of (de-sprouted) seed tubers at planting time of four cultivars stored at different temperatures (in colours) in three cycles. Error bars indicate ± standard deviation based on three blocks

**
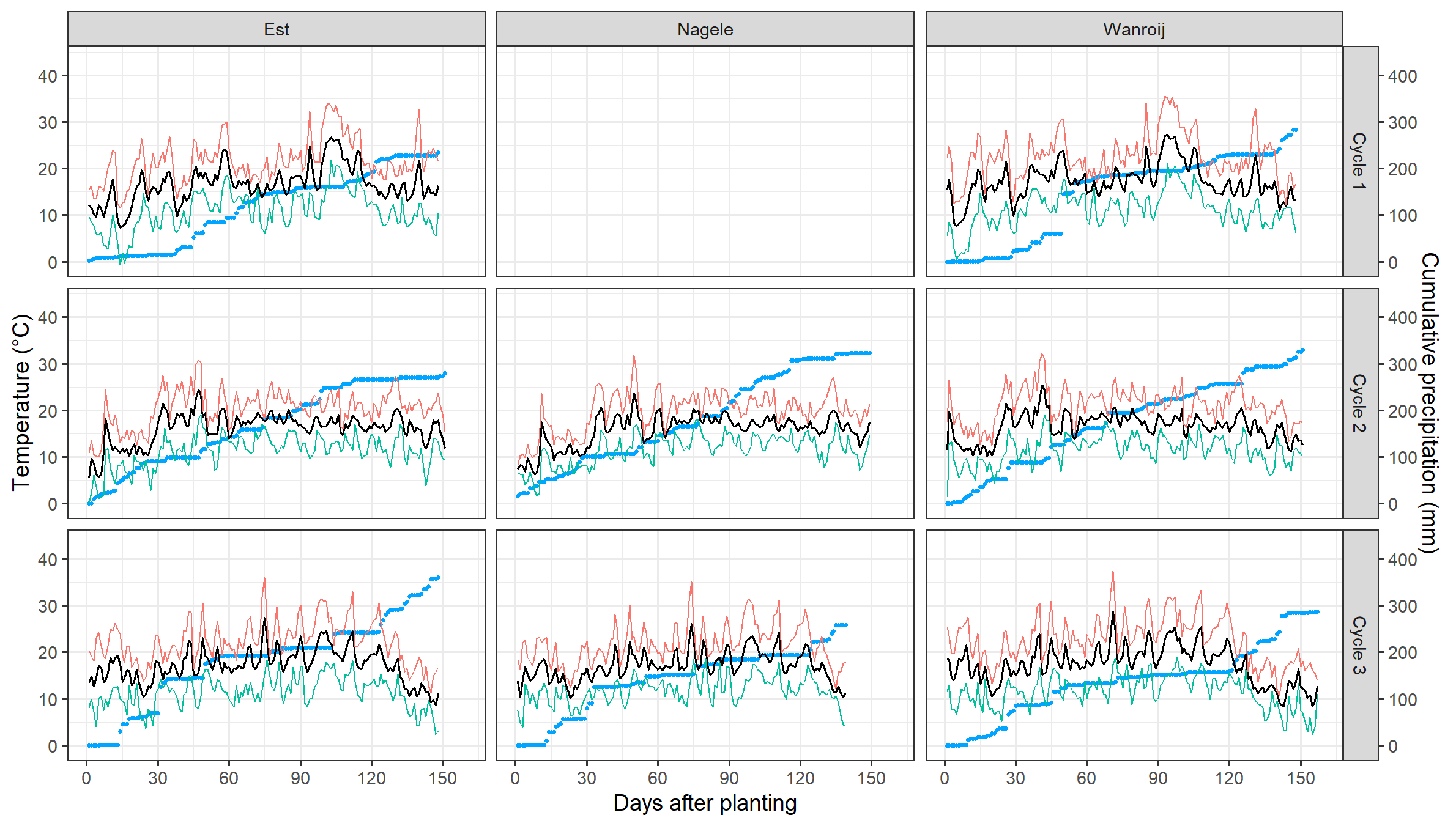
**

**Fig. S4** Daily average temperature (˚C, in black), daily maximum temperature (˚C, in red), daily minimum temperature (˚C, in green), and cumulative precipitation (mm, in blue) from planting to harvest at three sites in three cycles. Daily temperatures were calculated as the mean hourly temperatures in a day

**
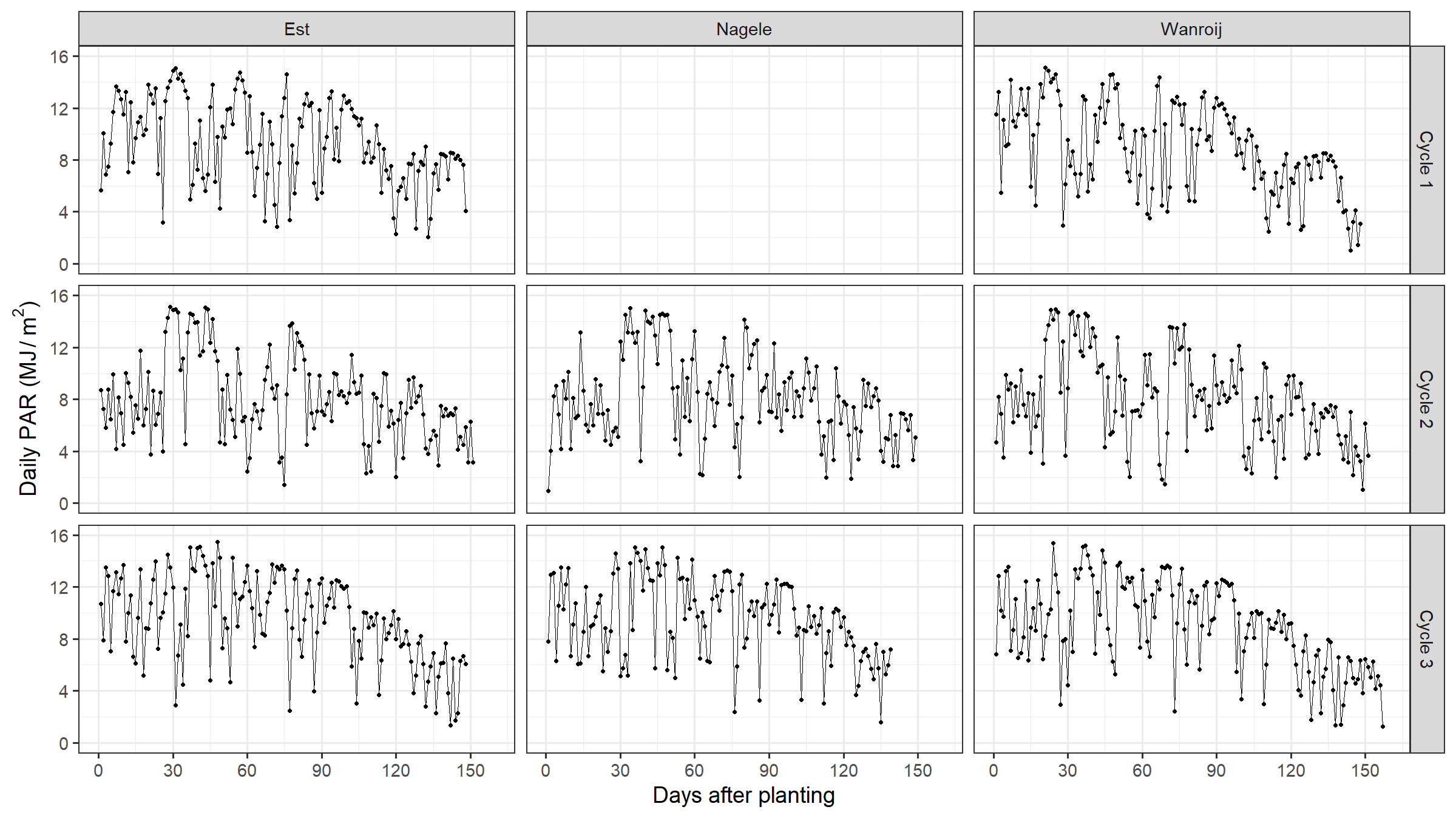
**

**Fig. S5** Daily incident photosynthetic active radiation (PAR) from planting to harvest at three sites in three cycles


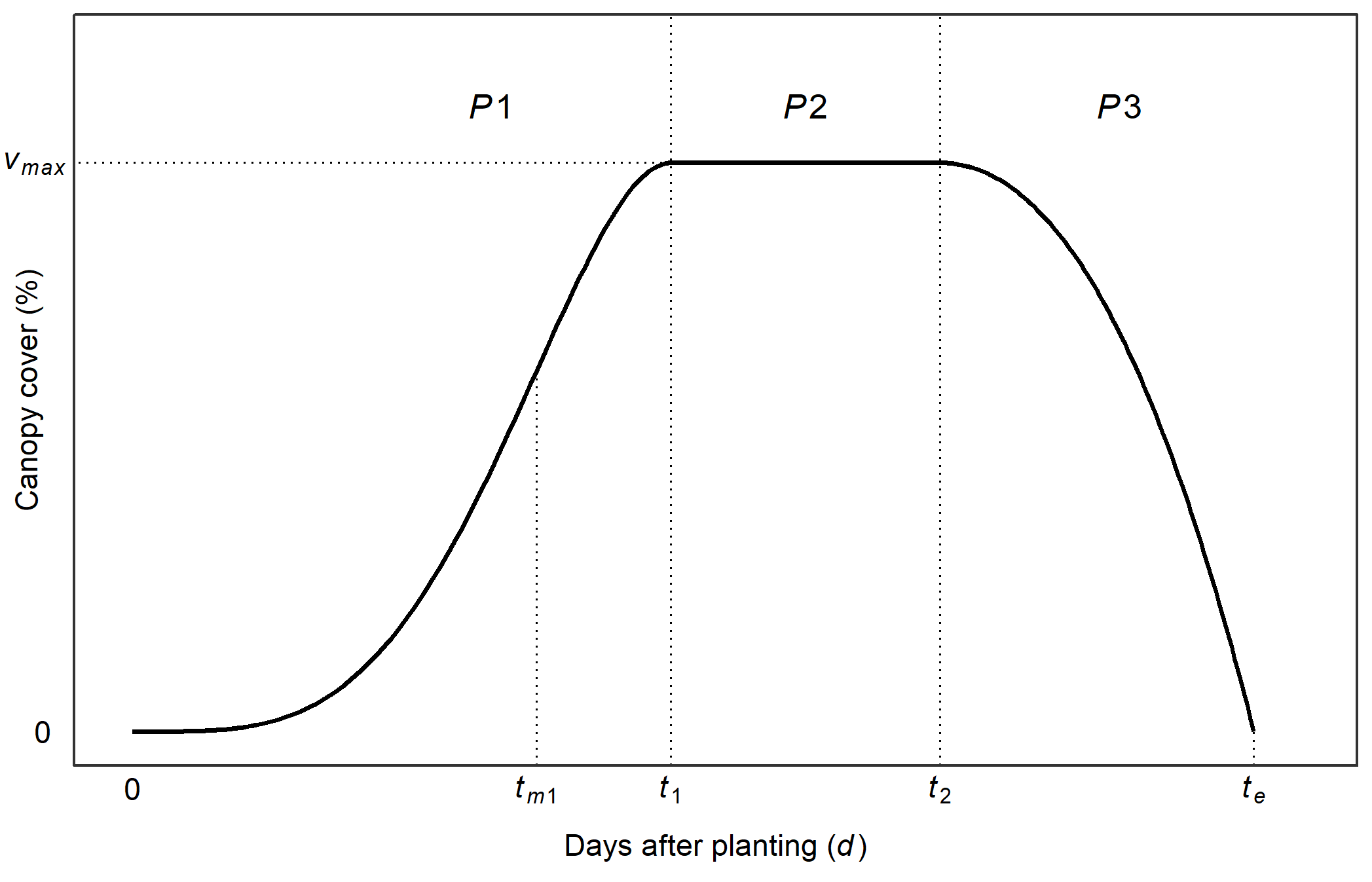


**Fig. S6** Temporal course of canopy cover development from time 0 to *t_e_* described by the model of (Khan et al., 2019). P1 (canopy build-up phase), P2 (maximum canopy cover phase), and P3 (canopy decline phase)


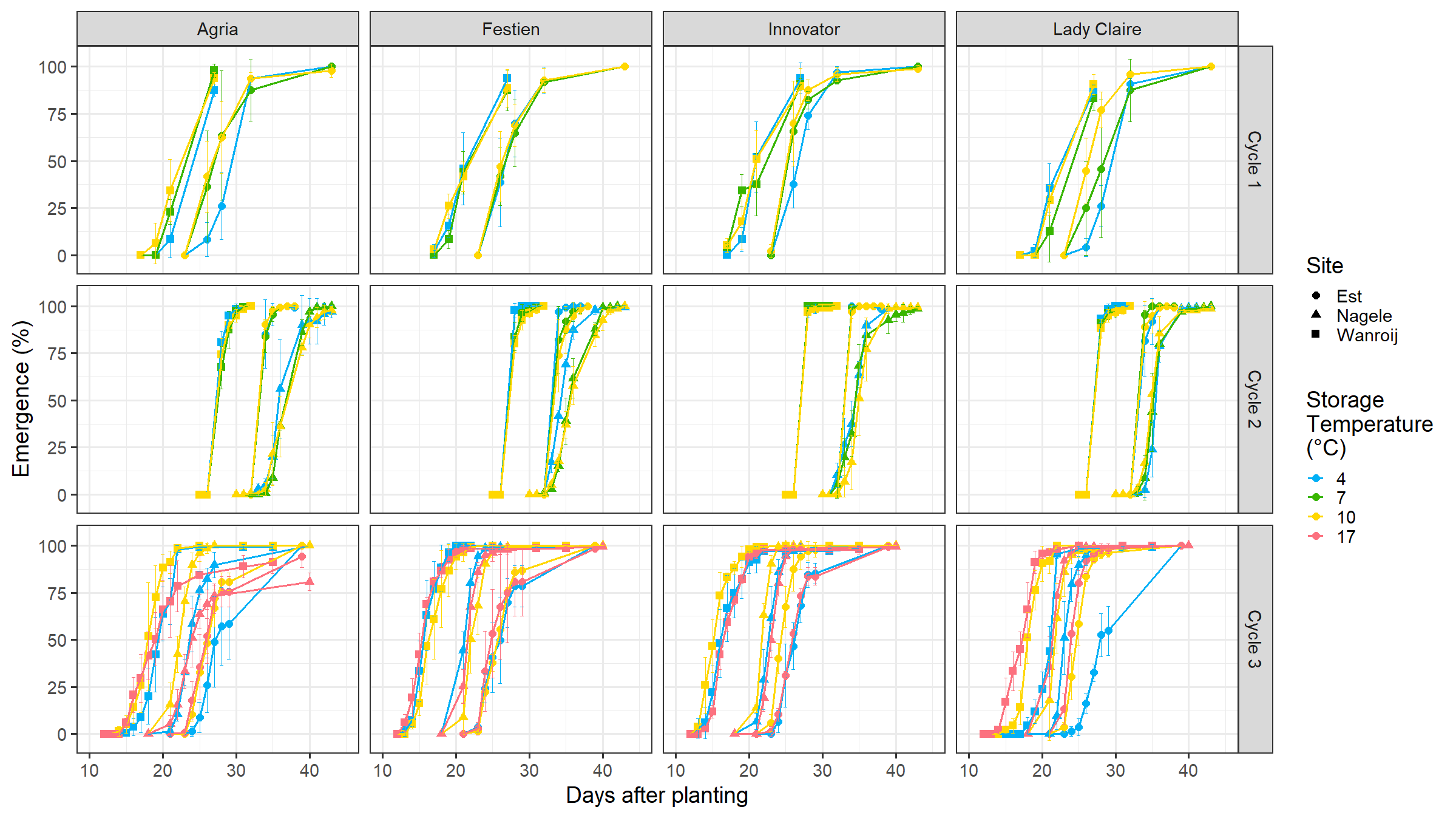


**Fig. S7** Percentage of observed emerged plants of four cultivars stored at different temperatures (in colours) against days after planting at three sites (in marker shapes) in three cycles. Error bars indicate ± standard deviation based on three blocks

**
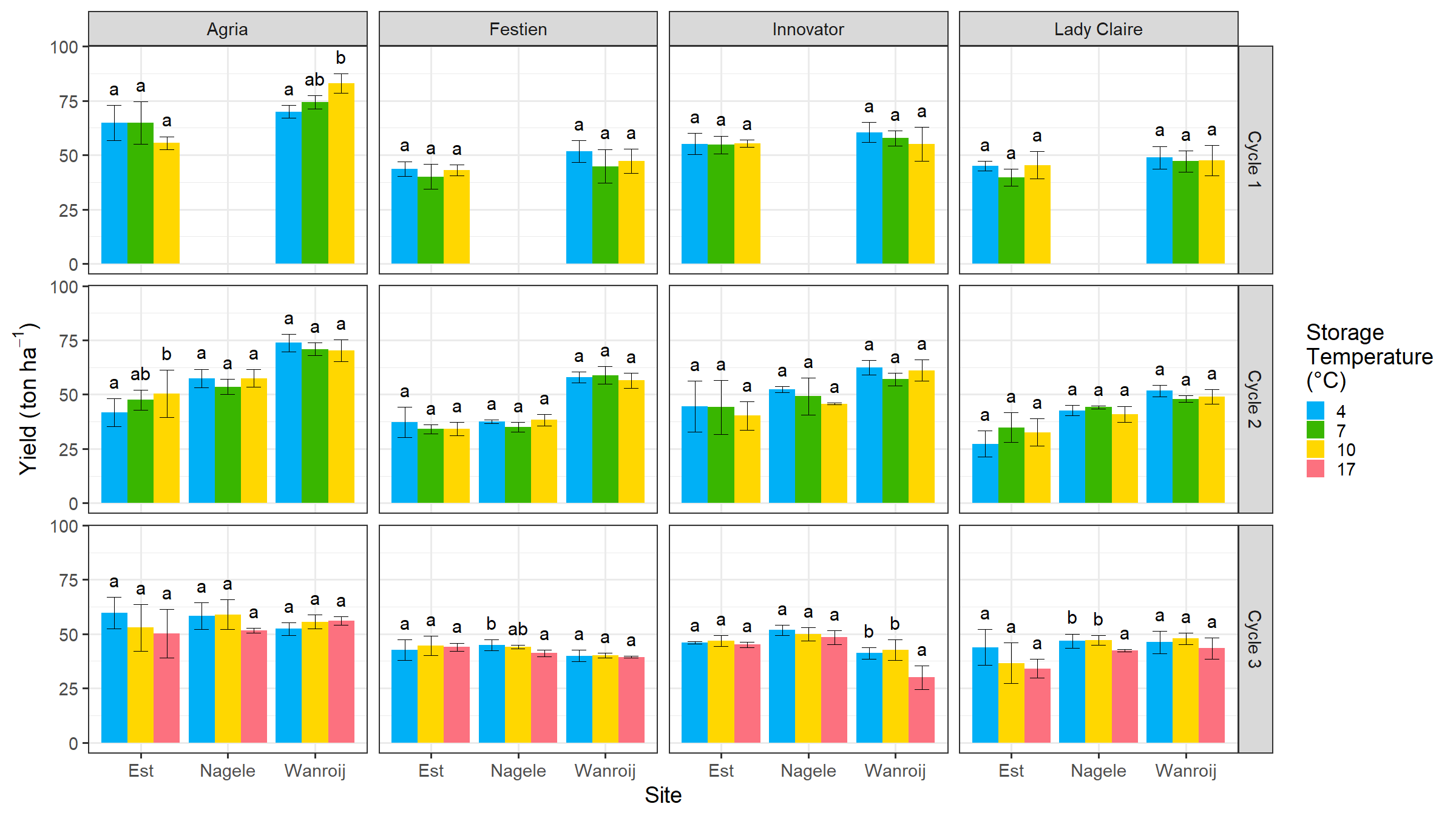
**

**Fig. S8** Final yield (ton ha^-1^) from planted seed tubers of four cultivars stored at different temperatures (in colours) at three sites in three cycles. Error bars indicate ± standard deviation based on three blocks. No overlap in letters indicates a significant difference between storage temperatures of each cycle × site × cultivar combination in post-hoc tests


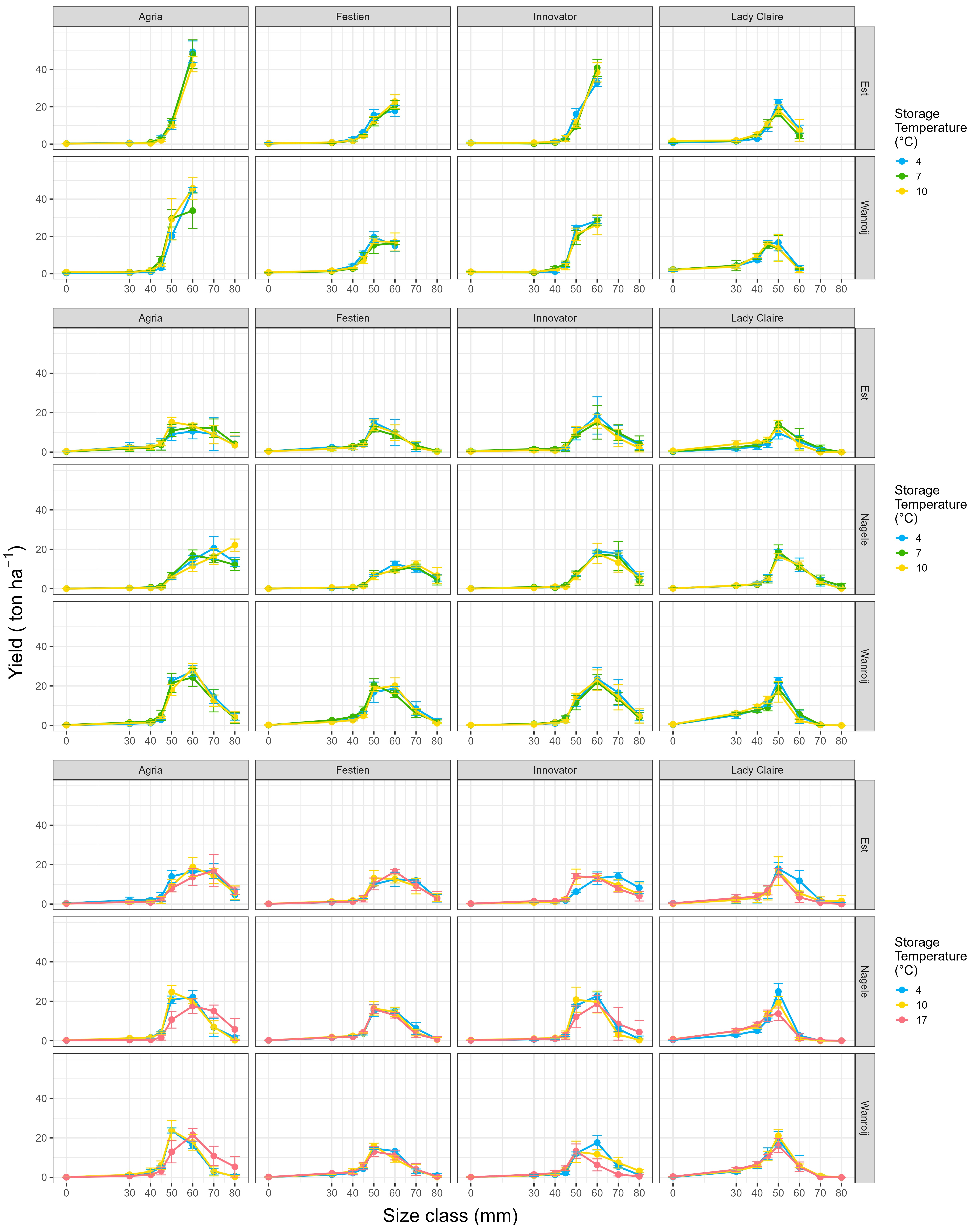


**Fig. S9** Final yield (ton ha^-1^) of harvested tubers between each and the subsequent size class (mm) of four cultivars stored at different temperatures (in colours) at three sites in three cycles. E.g. ‘80 mm’ indicates sizes of ‘80 mm’ and ‘80 mm +’. Error bars indicate ± standard deviation based on three blocks
